# Fatty acid desaturase 2 determines the lipidomic landscape and steroidogenic function of the adrenal gland

**DOI:** 10.1126/sciadv.adf6710

**Published:** 2023-07-21

**Authors:** Anke Witt, Ivona Mateska, Alessandra Palladini, Anupam Sinha, Michele Wölk, Akiko Harauma, Nicole Bechmann, Christina Pamporaki, Andreas Dahl, Michael Rothe, Irakli Kopaliani, Christian Adolf, Anna Riester, Ben Wielockx, Stefan R. Bornstein, Matthias Kroiss, Mirko Peitzsch, Toru Moriguchi, Maria Fedorova, Michal Grzybek, Triantafyllos Chavakis, Peter Mirtschink, Vasileia Ismini Alexaki

**Affiliations:** ^1^ Institute for Clinical Chemistry and Laboratory Medicine, Faculty of Medicine and University Hospital Carl Gustav Carus, Technische Universität Dresden, Dresden, 01307, Germany.; ^2^ Department of Physiology, Faculty of Medicine, Technische Universität Dresden, Dresden, 01307, Germany.; ^3^ Center of Membrane Biochemistry and Lipid Research, Faculty of Medicine, Technische Universität Dresden, Dresden, 01307, Germany.; ^4^ Paul Langerhans Institute Dresden of the Helmholtz Centre Munich at the University Hospital Carl Gustav Carus, Technische Universität Dresden, Dresden, 01307, Germany.; ^5^ German Center for Diabetes Research (DZD e.V.), Neuherberg, 85764, Germany.; ^6^ School of Life and Environmental Science, Azabu University, 1-17-71 Fuchinobe, Sagamihara, Kanagawa, 252-5201, Japan.; ^7^ Department of Internal Medicine III, University Hospital Carl Gustav Carus, Technische Universität Dresden, Dresden, 01307, Germany.; ^8^ DRESDEN-Concept Genome Center, Center for Molecular and Cellular Bioengineering, Technische Universität Dresden, Dresden, 01307, Germany.; ^9^ Lipidomix GmbH, Berlin, Germany.; ^10^ Department of Internal Medicine IV, University Hospital Munich, Ludwig-Maximilians-Universität München, Munich, 80336, Germany.; ^11^ Department of Internal Medicine I, Division of Endocrinology and Diabetes, University Hospital, University of Wuerzburg, Wuerzburg, 97080, Germany.; ^12^ Centre for Cardiovascular Science, Queen’s Medical Research Institute, University of Edinburgh, Edinburgh, EH16 4TJ, UK.

## Abstract

Corticosteroids regulate vital processes, including stress responses, systemic metabolism, and blood pressure. Here, we show that corticosteroid synthesis is related to the polyunsaturated fatty acid (PUFA) content of mitochondrial phospholipids in adrenocortical cells. Inhibition of the rate-limiting enzyme of PUFA synthesis, fatty acid desaturase 2 (FADS2), leads to perturbations in the mitochondrial lipidome and diminishes steroidogenesis. Consistently, the adrenocortical mitochondria of *Fads2^−/−^* mice fed a diet with low PUFA concentration are structurally impaired and corticoid levels are decreased. On the contrary, FADS2 expression is elevated in the adrenal cortex of obese mice, and plasma corticosterone is increased, which can be counteracted by dietary supplementation with the FADS2 inhibitor SC-26192 or icosapent ethyl, an eicosapentaenoic acid ethyl ester. In humans, *FADS2* expression is elevated in aldosterone-producing adenomas compared to non-active adenomas or nontumorous adrenocortical tissue and correlates with expression of steroidogenic genes. Our data demonstrate that FADS2-mediated PUFA synthesis determines adrenocortical steroidogenesis in health and disease.

## Teaser

FADS2 determines the lipidome and steroidogenic function of the adrenal gland.

## INTRODUCTION

The corticosteroids aldosterone and cortisol are produced in the *zona glomerulosa* and *zona fasciculata* of the adrenal cortex, respectively. Aldosterone increases the blood volume and pressure, while cortisol mediates stress responses, including regulation of glucose and lipid metabolism (*1*, *2*). Excess production of aldosterone in primary hyperaldosteronism can cause resistant hypertension and hypokalemia (*1*). Hypercortisolismus leads to severe comorbidities, including central obesity, dyslipidemia, and hypertension collectively termed Cushing syndrome (*3*). Obesity is also associated with increased aldosterone and glucocorticoid production, which exacerbates central obesity, hyperlipidemia, hypertension, and risk for cardiovascular disease (*4*–*8*). However, the underlying mechanisms of the increased corticoid production in obesity are not fully understood.

Aldosterone production is induced by angiotensin II through calcium signaling in the steroidogenic cells of the *zona glomerulosa* (*9*). Adrenocorticotropic hormone (ACTH), released by the pituitary, triggers cyclic adenosine 3′,5′-monophosphate signaling and induces glucocorticoid synthesis in the steroidogenic cells of the *zona fasciculata*. Corticosteroid synthesis requires the release of cholesterol from lipid droplets where it is stored in the form of cholesterol esters (CE) and its translocation into mitochondria via the steroidogenic acute regulatory (StAR) protein, which is the rate-limiting step of steroidogenesis. Once inside the mitochondria, cholesterol is used for steroidogenesis through a series of synthetic steps. CYP11A1 (P450scc)-mediated side-chain cleavage of cholesterol produces pregnenolone which is then converted to progesterone by the nicotinamide adenine dinucleotide–dependent 3β-hydroxysteroid dehydrogenase (3β-HSD). CYP21 catalyzes the NADPH-dependent hydroxylation of progesterone and 17a-hydroxyprogesterone to 11-deoxycorticosterone and 11-deoxycortisol, respectively. In the *zona glomerulosa*, CYP11B2 catalyzes the production of aldosterone from 11-deoxycorticosterone. In the *zona fasciculata*, CYP11B1 catalyzes the conversion of 11-deoxycortisol to cortisol in humans and 11-deoxycorticosterone to corticosterone in mice (*2*, *10*).

We here investigated the impact of mitochondrial membrane lipids on steroidogenesis in adrenocortical cells. We show that inhibition of fatty acid desaturase 2 (FADS2), the rate-limiting enzyme of polyunsaturated fatty acid (PUFA) synthesis, transforms the mitochondrial lipidome, inhibits cholesterol import, and diminishes steroidogenesis. Accordingly, FADS2 deficiency in mice impairs mitochondrial structure in adrenocortical cells and reduces corticosterone and aldosterone production. Conversely, FADS2 expression is up-regulated in the adrenal glands of obese mice and in aldosterone-producing adenomas compared to non-active adenomas (producing low amounts of aldosterone) and nontumorous adrenocortical tissue of patients, while FADS2 inhibition reduces corticoid levels in obese mice. These findings demonstrate that FADS2 determines mitochondrial structure and function and highlight the crucial role of FADS2 in adrenocortical steroidogenesis.

## RESULTS

### FADS2 determines the mitochondrial lipidome and is required for steroidogenesis in adrenocortical cells

**Fig. 1.**
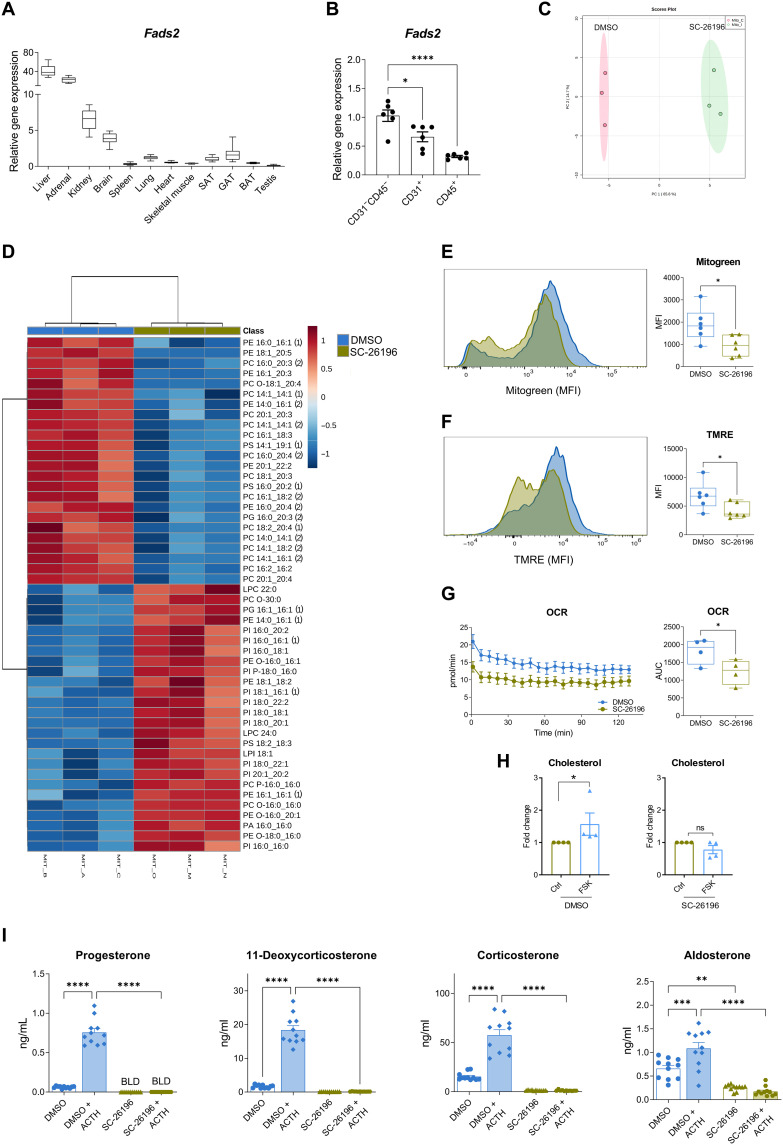
FADS2 determines the mitochondrial lipidome and is required for steroidogenesis in adrenocortical cells. (**A**) Relative *Fads2* expression in different tissues of 8-week-old WT C57BL6J mice determined by qPCR using *Tbp* as a housekeeping gene. Data are shown as mean 2^Δ*C*t^ (*n* = 8). BAT, brown adipose tissue; SAT, subcutaneous adipose tissue; GAT, gonadal adipose tissue. (**B**) Relative *Fads2* expression in CD31^−^CD45^−^, CD31^+^, and CD45^+^ cell populations of the mouse adrenal cortex. Expression of *Fads2* in CD31^−^CD45^−^ cells was set to 1. 18*S* was used as an internal control (*n* = 6). (**C** and **D**) PCA of the phospholipidome (C) and heatmap showing the differentially regulated lipid species (D) of mitochondrial fractions of NCI-H295R cells treated for 18 hours with SC-26196 (10 μM) or equal amount of DMSO (*n* = 3, one of two experiments). (**E** and **F**) Mitogreen (E) and TMRE (F) staining in primary adrenocortical cells treated for 18 hours with SC-26196 (10 μM) or DMSO (*n* = 6). MFI, mean fluorescence intensity. (**G**) OCR measurement of primary adrenocortical cells treated for 18 hours with SC-26196 or DMSO (*n* = 4). AUC, area under curve (**H**) Cholesterol levels determined by LC-MS/MS in mitochondrial fractions of NCI-H295R cells treated for 18 hours with SC-26196 or DMSO and stimulated or not with forskolin (FSK; 1 μM) for 15 min. Results of three independent experiments are shown here. (**I**) Progesterone, 11-deoxycorticosterone, corticosterone, and aldosterone levels were determined by LC-MS/MS in supernatants of primary adrenal cell cultures treated for 18 hours with SC-26196 or DMSO and then stimulated or not for 1 hour with ACTH (10 ng/ml) in the presence of SC-26196 or DMSO. The cell culture medium was changed before ACTH treatment (*n* = 12). Data in (B), (H), and (I) are shown as mean ± SEM, *P < 0.05; **P < 0.01; ***P < 0.001; ****P < 0.0001.

As key steps of steroidogenesis take place in mitochondria (*10*), we first interrogated to which extent the lipid composition of mitochondria affects steroidogenesis in adrenocortical cells. We chose Δ-6-desaturase FADS2 as a target to modulate mitochondrial lipid composition in adrenocortical cells, since it is highly expressed in the adrenal gland compared to other tissues and organs (Fig. 1A and fig. S1A), and its expression is higher in steroidogenic cells (CD31^−^CD45^−^) compared to endothelial cells (CD31^+^) and leukocytes (CD45^+^) of the adrenal cortex (Fig. 1B). The adrenal gland and the liver were the organs with the highest FADS2 mRNA and protein expression among many different tested organs and tissues (Fig.1A and fig. S1A). Moreover, FADS2 is the rate-limiting enzyme in the synthesis of PUFAs, which are important components of functional and storage lipids (*11*, *12*). FADS2 catalyzes the conversion of the omega-6 linoleic acid (18:2 n-6, LA) to γ-linolenic acid (18:3 n-6, GLA) and the omega-3 α-linolenic acid (18:3 n-3, ALA) to stearidonic acid (18:4 n-3) (fig. S1B) (*13*).

We treated NCI-H295R adrenocortical carcinoma cells for 18 hours with the FADS2 inhibitor SC-26196 and analyzed the lipidome of mitochondrial fractions [identified by succinate dehydrogenase B (SDHB) expression] by liquid chromatography–tandem mass spectrometry (LC-MS/MS) (fig. S1C). FADS2 inhibition reprogramed the mitochondrial lipidome, as shown by principal components analysis (PCA) (Fig. 1C) and strongly altered the mitochondrial lipid composition at individual species level (Fig. 1D). The abundance of phosphatidylcholines (PC) and phosphatidylethanolamines (PE) containing PUFA acyl chains, such as PC 16:0_20:4, PC 20:1_20:3, PC 20:1_20:4, PE 16:0_20:4, PE 18:1_20:5, and PE 16:1_20:3, was reduced in SC-26196–treated cells, while conversely lipids with saturated or monounsaturated acyl chains, such as PE 14:0_16:1, PE 16:1_16:1, phosphatidylinositol (PI) PI 16:0_16:0, PI 16:0_16:1, PI 16:0_18:1, PI 18:1_16:1, PI 18:0_18:1, PI 18:0_20:1, PI 18:0_22:1, and phosphatidylglycerol (PG) PG 16:1_16:1, were increased (Fig. 1D). Cardiolipins, a unique component of the inner mitochondrial membrane (IMM), remained unaffected (not shown).

PC and PE are major lipid components of mitochondrial membranes, and therefore, changes in their consistency are likely to influence mitochondrial physiology (*14*, *15*). FADS2 inhibition decreased the mitochondrial load, mitochondrial membrane potential, and oxygen consumption rate (OCR) in primary adrenocortical cells (Fig. 1, E to G). Electron transport chain–dependent mitochondrial bioenergetics are intimately linked with and required for steroidogenesis (*10*, *16*–*19*). Hence, the observed reduction of the mitochondrial membrane potential (Fig. 1F) and OCR (Fig. 1G) is expected to lead to reduced steroidogenesis. Along this line, we asked whether cholesterol transport into mitochondria, which is the rate-limiting step of steroidogenesis, is affected by FADS2 inhibition. Cholesterol import into mitochondria involves the interaction between StAR, voltage-dependent anion channel (VDAC), and translocator protein, 18 kDa (TSPO) (*10*). StAR shuttles from the cytoplasm to the outer mitochondrial membrane (OMM), transferring cholesterol to a complex, which is assembled by VDAC and TSPO at the OMM-IMM contact sites, called transduceosome (*10*, *19*). In adrenocortical steroidogenic cells, cholesterol taken up from circulating lipoproteins or synthesized in the endoplasmic reticulum (ER), is mainly stored in the form of CE in lipid droplets, from where it is transferred by StAR and the transduceosome into the mitochondria for pregnenolone synthesis, the first steroid to be synthesized in the steroidogenic cascade (*19*). To examine whether FADS2 inhibition affects cholesterol uptake in the mitochondria, we analyzed the cholesterol content of mitochondrial (SDHB positive) fractions of NCI-H295R cells treated or not with SC-26196 upon stimulation with forskolin (fig. S1C). Forskolin treatment triggered a strong increase in mitochondrial cholesterol levels in control but not SC-26196–treated cells (Fig. 1H). In accordance, cholesterol and CE accumulated in the lipid droplet [hormone-sensitive lipase (HSL) positive] fractions in SC-26196–treated cells (fig. S1, C and D).

Reduced mitochondrial bioenergetics (Fig. 1, F and G) and cholesterol mitochondrial import (Fig. 1H) in SC-26196–treated cells are expected to lead to impaired steroidogenesis. To verify this, we analyzed steroid hormone production by LC-MS/MS in primary adrenal cell cultures treated with SC-26196 and subsequently stimulated with ACTH. ACTH efficiently increased progesterone, 11-deoxycorticosterone, corticosterone, and aldosterone production, while FADS2 inhibition abolished their production (Fig. 1I), without affecting cell viability (fig. S1E). FADS2 inhibition also reduced progesterone and 11-deoxycortisol production in forskolin-treated NCI-H295R cells (fig. S1F). The effect of FADS2 inhibition on steroidogenesis was not due to decreased mRNA expression of *StAR* or steroidogenic enzymes, Cytochrome P450 Family 11 Subfamily A Member 1 (*Cyp11a1*, cholesterol side-chain cleavage enzyme), *3*β*-Hsd2*, *Cyp11b1*, or *Cyp11b2*; their expression was increased upon FADS2 inhibition, perhaps as a compensatory response to low steroid hormone synthesis (fig. S1G). These data indicate that increased expression of steroidogenic enzymes is not sufficient for successful steroidogenesis when FADS2 is blocked, thereby underscoring the importance of FADS2-dependent PUFA synthesis for steroidogenesis. In accordance, FADS2 overexpression (fig. S1, H and I) increased 11-deoxycorticosterone, cortisol, and aldosterone production in forskolin-treated NCI-H295R cells (fig. S1J) substantiating the promoting effect of FADS2 on steroidogenesis.

### High-fat diet reprograms the adrenal lipidome and increases glucocorticoid production

**Fig. 2.**
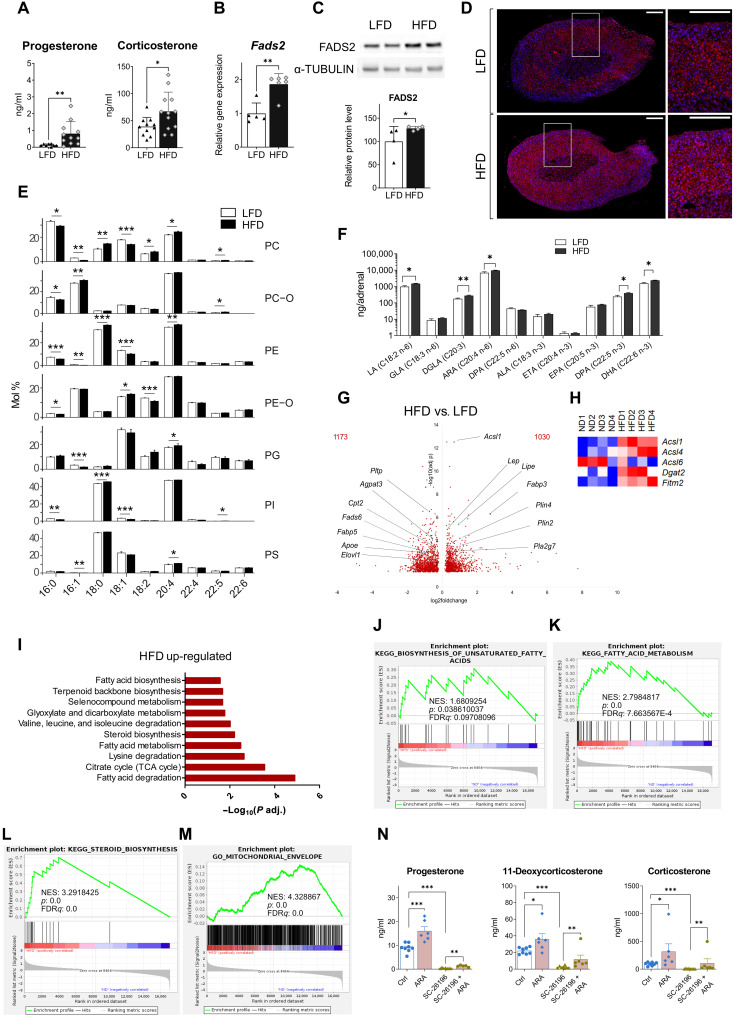
HFD reprograms the adrenal lipidome and increases glucocorticoid production. (**A**) Progesterone and corticosterone plasma levels in mice fed for 20 weeks a LFD or HFD (*n* = 8 to 12). (**B** and **C**) *Fads2* mRNA expression (B) and protein expression (C) in adrenal glands of LFD and HFD mice (*n* = 4 to 6). (**D**) Immunofluorescence for FADS2 (red) and 4′,6-diamidino-2-phenylindole (DAPI; blue) in adrenal glands of LFD- and HFD mice (1 of 5 mice per condition). Scale bars, 200 μm, zoomed-in inserts are shown at the right. (**E**) Acyl chain profile of nonstorage lipids analyzed by shotgun lipidomics. Lipids in adrenal glands from lean and obese mice were grouped according to the acyl chain within each lipid class (mol % is relative to the lipid class). Only the features with a mean abundance >5 mol % are shown, mean mol % ± SD is shown (*n* = 7 to 8). (**F**) Acyl chain isomers in phospholipids in adrenal glands of LFD and HFD mice determined by HPLC-MS (*n* = 5 to 6). (**G** to **M**) RNA-seq in the adrenal cortex of LFD and HFD mice (*n* = 4 mice per group). Volcano plot showing differentially expressed genes in HFD versus LFD mice (G). Heatmap of differentially expressed genes involved in fatty acid processing (P adjusted < 0.05) (H). EGSEA of RNA-seq data in the adrenal cortex of HFD versus LFD mice showing 10 among most up-regulated metabolic pathways (KEGG database) (I). GSEA for genes involved in biosynthesis of unsaturated fatty acids (J), fatty acid metabolism (K), steroid biosynthesis (L), and mitochondrial envelope formation (M). NES, normalized enrichment score; FDR, false discovery rate. (**N**) Progesterone, 11-deoxycorticosterone, and corticosterone levels in supernatants of primary adrenal cell cultures treated for 18 hours with SC-26196 (10 μM) or DMSO (Ctrl) in the presence or absence of ARA (150 μM) (*n* = 6 to 8). Data in (A) to (C) are shown as mean ± SD and in (F) and (N) as mean ± SEM. *P < 0.05; **P < 0.01; ***P < 0.001.

Next, we asked whether FADS2 could drive increased adrenocortical hormone production in vivo. As an experimental model we used high-fat diet (HFD)–induced obesity in mice, which was previously shown to lead to increased corticosterone and aldosterone production (*5*). Wild-type (WT) C57BL6/J mice were fed for 20 weeks a low-fat diet (LFD; 10% kcal deriving from fat) or a HFD (60% kcal deriving from fat). HFD feeding substantially increased the body weight of the animals (fig. S4A), as previously reported in a large number of studies (*20*, *21*). As expected, progesterone and corticosterone plasma levels were elevated in HFD compared to LFD mice (Fig. 2A). ACTH plasma levels were also increased in obese mice (fig. S2A), standing in accordance with the notion that chronic inflammation and dysmetabolism may lead to chronic activation of the hypothalamic-pituitary-adrenal (HPA) axis (*22*, *23*). Adrenal gland weights did not change with obesity (fig. S2B). We next interrogated the lipidomic changes in the adrenal glands of obese mice, and whether these changes may contribute to the increased steroidogenesis in an autonomous fashion.

FADS2 expression was elevated at mRNA and protein level in the adrenal glands of HFD-fed mice (Fig. 2, B to D), while its expression did not change in other organs, such as the liver or the brain (fig. S2C). To understand the role of FADS2 in regulating the adrenocortical lipidome, we investigated the adrenal gland lipidome by shotgun lipidomics and revealed reprograming of the whole lipidome, i.e., both nonstorage lipids and storage lipids upon HFD feeding (fig. S2D). Nonstorage, i.e., membrane lipids include phospholipids and their ether-linked versions [PC, PE, PC O-, PE O-, PI, PG, phosphatidylserine (PS), and phosphatidate (PA)], lysolipids [lyso-phosphatidate (LPA), lyso-phosphatidylcholine (LPC), phosphatidylethanolamine (LPE), LPE O-, lyso-phosphatidylglycerol (LPG), lyso-phosphatidylinositol (LPI), and lyso-phosphatidylserine (LPS)], sphingomyelin (SM), diacylglycerol (DAG), cardiolipins (CL), ceramides, and hexosylceramides (HexCer). Storage lipids comprise CE and triacylglycerol (TAG) (*24*). Whole and nonstorage lipidomes of adrenal glands from lean and obese mice segregated along the first principal component (PC1), and the topology of the samples was notably similar, while storage lipids regrouped with statistical significance along the second principal (PC2) (fig. S2D), suggesting that nonstorage lipids were mainly responsible for the grouping of the whole lipidome according to the diet.

Yet, storage lipids (TAG and CE) constituted the largest portion of the identified lipids in adrenal glands (86.98 ± 5.43% in LFD mice and 90.12 ± 1.93% in HFD mice), while nonstorage lipids accounted for 13.02 ± 5.22% and 9.87 ± 1.89% of all identified lipids in the adrenal glands of mice under LFD and HFD, respectively (table S1). The total amounts of storage and nonstorage lipids did not substantially differ in the adrenal glands of lean and obese mice (table S1). Likewise, lipid classes did not significantly differ between the adrenal glands of lean and obese mice (table S2). CE as well as non-esterified cholesterol were also not significantly increased in the adrenal glands of obese mice (table S2).

Our data show that it is not the lipid amount but the total acyl chain length and degree of unsaturation of storage and nonstorage lipids that substantially changed with obesity. TAG species with 50 to 54 carbon atoms (fig. S2E) and two to four acyl chain double bonds (fig. S2F) were highly abundant (mol % > 10). Adrenal glands of HFD mice exhibited a significant increase in long (with a total acyl chain length of 54 carbon atoms) and unsaturated (with four, five, and more than six double bonds) TAG species. In contrast, adrenal glands from LFD-fed mice showed a larger amount of TAG species containing shorter acyl chains (with 48 and 50 carbon atoms) with one or two double bonds (fig. S2, E and F). To determine whether the diet affected the overall mean weighted length and unsaturation level of lipids, we calculated the double bond index (DBI) and the length index (LI). Storage lipids in the adrenal glands of obese mice showed a significantly higher DBI compared to lean mice (table S3). Hence, upon HFD feeding, TAG in the adrenal glands became overall more unsaturated.

Most nonstorage lipids contained a cumulative of 34 to 38 carbon atoms (fig. S2G) and four double bonds in their acyl chains (fig. S2H). Similarly to what was found for TAG, adrenal glands from HFD mice showed a significant increase in longer and more unsaturated (with four double bonds) nonstorage lipids, while shorter and mono-unsaturated nonstorage lipid species were more abundant in the adrenal glands of LFD-fed mice (fig. S2, G and H). In accordance, membrane lipids in the adrenal glands of HFD-fed mice had significantly higher DBI and LI compared to LFD-fed mice (table S3), indicating that nonstorage (membrane) lipids were longer and more unsaturated in the adrenal glands of obese mice.

Notably, 20:4 was one of the most abundant acyl chains in PC, PC O-, PE, PE O-, PG, and PI, and the amount of PC, PE, and PG lipids containing 20:4 was higher in the adrenal glands of obese compared to lean mice (Fig. 2E). In contrast, long-chain PUFAs other than 20:4, such as 22:4, 22:5, and 22:6, were less abundant (Fig. 2E). Accordingly, the phospholipids PC 18:0_20:4 and PE 18:0_20:4 were highly abundant and higher in the adrenal glands of HFD mice (fig. S2, I and J).

Isomer analysis showed that arachidonic acid (ARA; 20:4 n-6, *all-cis-*5,8,11,14-eicosatetraenoic acid) but not eicosatetraenoic acid (ETA; 20:4 n-3, *all*-cis-8,11,14,17-eicosatetraenoic acid) was the abundant 20:4 isomer form (Fig. 2F), in agreement with previous reports showing very little ETA in the tissue lipidome (*25*). Furthermore, docosapentaenoic acid (DPA; 22:5 n-3) and docosahexaenoic acid (DHA 22:6 n-3) were more abundant in adrenal gland phospholipids of obese mice, while eicosapentaenoic acid (EPA; 20:5 n-3) was not (Fig. 2F). Given that FADS2 is the rate-limiting enzyme for ARA, DPA, and DHA synthesis (fig. S1B), the increased amounts of these phospholipidic acyl chains in the adrenal glands of HFD mice could be the result of an enhanced adrenal FADS2 expression in obese mice (Fig. 2, B to F).

To correlate the observed shift at the lipidome level with transcriptional changes, we performed RNA sequencing (RNA-seq) in the adrenal cortex of LFD and HFD mice that revealed 2203 differentially expressed genes, of which 1030 were up-regulated and 1173 were down-regulated by HFD compared to LFD feeding (Fig. 2G). Several genes involved in lipid metabolism were highly up-regulated, such as Acyl-coenzyme A synthetase long-chain family member 1 (*Acsl1*), Fatty acid binding protein 3 (*Fabp3*), Lipase e (*Lipe*), and Perilipin 4 (*Plin4*), or down-regulated, such as Phospholipid transfer protein (*Pltp*), *Fads6*, 1-Acylglycerol-3-phosphate O-acyltransferase 3 (*Agpat3*), or Carnitine palmitoyltransferase (*Cpt2*) in the adrenal cortex of HFD mice (Fig. 2G). *Fads2* was also up-regulated in the adrenal cortex of HFD- compared to LFD-fed mice (log_2_ fold change = 1.244, adjusted *P* value = 0.0117), as were several other genes involved in fatty acid processing, such as *Acsl4*, the most highly expressed *Acsl* isoform in the adrenal gland, Diacylglycerol O-acyltransferase 2 (*Dgat2*), and Fat storage–inducing transmembrane protein 2 (*Fitm2*) (Fig. 2H). Ensemble of Gene Set Enrichment Analysis (EGSEA) showed that fatty acid metabolism, steroid biosynthesis, and tricarboxylic acid (TCA) cycle pathways were among the most up-regulated pathways in the adrenal cortex of HFD mice (Fig. 2I). Similarly, Gene Set Enrichment Analysis (GSEA) also demonstrated significant positive enrichment of genes related to biosynthesis of unsaturated fatty acids, lipid metabolism, and steroid biosynthesis in the adrenal cortex of obese mice (Fig. 2, J to L, and fig. S2K). Also genes associated with mitochondrial biogenesis (fig. S2L), formation of the mitochondrial envelope (Fig. 2M), and mitochondrial function, including respiratory electron transport, oxidative phosphorylation, TCA cycle, and beta-oxidation (fig. S2, M to P), were all positively enriched in the adrenal cortex of HFD mice.

In summary, these findings demonstrate that HFD-induced obesity is associated with lipidomic transformation of the adrenal gland, particularly enhanced PUFA synthesis, increased FADS2 expression, and transcriptional changes promoting increased PUFA metabolism and mitochondrial bioenergetics in the adrenal cortex. A particularly abundant PUFA in adrenal PC and PE is ARA (Fig. 2, E and F). ARA was previously shown to promote steroidogenesis and potentially be required for cholesterol import into mitochondria (*26*, *27*). We found that FADS2 is required for the incorporation of ARA acyl chains in mitochondrial PC and PE (Fig. 1D). Hence, we tested whether ARA supplementation can counteract the inhibitory effect of FADS2 inhibition on steroidogenesis. ARA increased progesterone, 11-deoxycorticosterone, and corticosterone production in primary adrenocortical cells treated with SC-26196 (Fig. 2N). The fact that ARA only partially restored steroidogenesis could be due to insufficient lipidomic remodeling achieved by ARA supplementation under the used experimental conditions or may indicate the requirement of additional PUFAs for full restorage of steroidogenesis under FADS2 inhibition. Overall, these data imply that intra-adrenal FADS2-dependent ARA synthesis is required for adrenocortical steroidogenesis.

### Medulla-derived PUFAs promote adrenocortical steroidogenesis

**Fig. 3.**
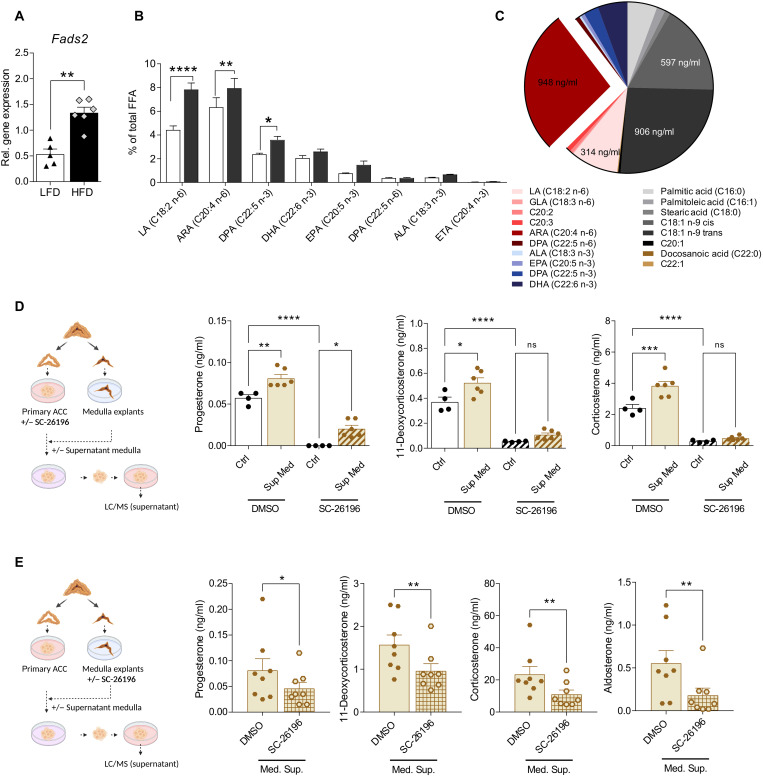
Medulla-derived PUFAs promote adrenocortical steroidogenesis. (**A**) Relative *Fads2* expression in the adrenal medulla of mice fed for 20 weeks a LFD or HFD (*n* = 5 to 6 mice per group). *Tbp* was used as a housekeeping gene. (**B**) FFA were measured in the adrenal medulla of mice fed for 20 weeks a LFD or HFD by HPLC-MS (*n* = 3 mice per group). Results are presented as % of total FFA. (**C**) FFA measured in the supernatants of adrenal medulla explants kept for 18 hours in 100 μl of medium. Results are presented as % of total FFAs (*n* = 4). (**D**) Adrenocortical cells were treated for 6 hours with SC-26196 (10 μM) or DMSO and then received medulla explant–conditioned or control medium for another 18 hours. Steroids were measured in the adrenocortical cell supernatant by LC-MS/MS (*n* = 4 to 6). (**E**) Adrenal medulla explants were treated for 6 hours with SC-26196 (10 μM) or DMSO, washed thoroughly, and left another 18 hours in culture. Their supernatant was then applied on adrenocortical cell cultures, and 8 hours later, adrenocortical cell supernatants were collected and analyzed by LC-MS/MS (*n* = 8). Data in (A), (B), (D), and (E) are shown as mean ± SEM. *P < 0.05; **P < 0.01; ***P < 0.001; ****P < 0.0001. ns, not significant.

The adrenal gland consists of the medulla and the cortex. Early reports showed that chromaffin (medullary) cells may release ARA (*28*). We asked whether FADS2-mediated PUFA synthesis in the medulla could influence adrenocortical steroidogenesis. *Fads2* expression in the adrenal medulla increased with obesity (Figs. 2D and 3A). In addition, free LA (C18:2 n-6) and ARA were increased in the medulla of obese compared to lean animals (Fig. 3B). In addition, free ARA was the most abundant free fatty acid (FFA) in the supernatant of medulla organ cultures (Fig. 3C). Together with our finding that ARA supplementation increased steroidogenesis in adrenocortical cells (Fig. 2N), these data suggest that ARA may mediate a medulla-cortex interaction promoting adrenocortical steroidogenesis.

To investigate how PUFAs from chromaffin cells may affect steroidogenesis in adrenocortical cells, we applied supernatants from medulla explants onto primary adrenocortical cell cultures. Medulla-conditioned supernatants increased progesterone, 11-dehydrocorticosterone, and corticosterone production in primary adrenocortical cultures compared to control medium (Fig. 3D). This effect was attenuated when medulla explants were treated with the FADS2 inhibitor SC-26196, underscoring the importance of medullar PUFA synthesis in the medulla-cortex interaction (Fig. 3E). However, treatment of adrenocortical cells with SC-26196 diminished steroid synthesis in cells receiving medulla-conditioned medium (Fig. 3D), suggesting that despite the fact that adrenocortical cells may take up medulla-deriving PUFA, it is mainly endogenous synthesis of PUFA in adrenocortical cells, which is required for steroidogenesis.

### FADS2 inhibition reduces corticosteroid levels in obese mice

**Fig. 4.**
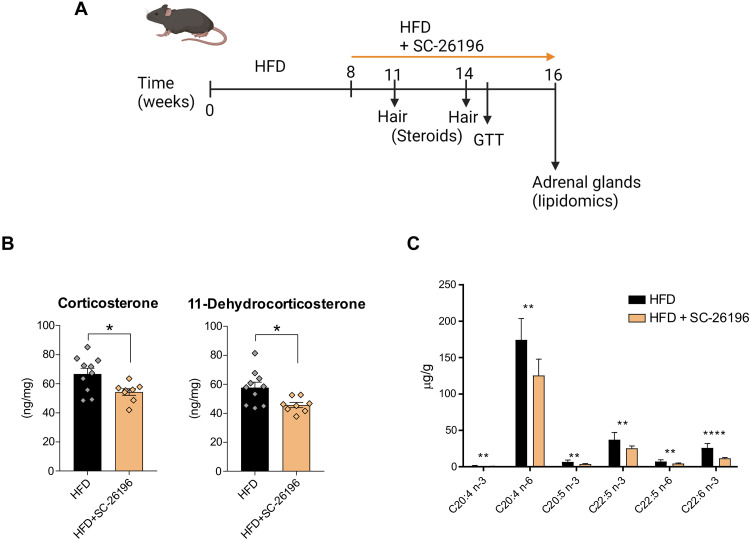
FADS2 inhibition reduces corticosteroid levels in obese mice. (**A**) Schematic representation of the experimental setup. (**B**) Corticosterone and 11-dehydrocorticosterone levels were determined by LC-MS/MS at feeding week 14 in hair of HFD mice receiving or not for 6 weeks SC-26196. Steroid concentrations were normalized to hair weight (B). (**C**) FFA were measured by HPLC-MS in the adrenal glands of mice fed a HFD with or without SC-26196 (*n* = 8 to 10 mice per group). Data are presented as mean ± SEM, *P < 0.05; **P < 0.01; ****P < 0.0001.

To validate the role of FADS2 in adrenocortical steroidogenesis in obesity, we treated HFD mice with the FADS2 inhibitor SC-26196 and assessed corticosteroid production. WT C57BL/6J mice were fed for 8 weeks a HFD and another 8 weeks with HFD containing or not SC-26196 (0.625 g/kg; corresponding to approximately 100 mg/kg of animal weight) (Fig. 4A). We decided to determine corticosteroid levels at two different time points, i.e., 3 and 6 weeks after SC-26196 treatment start, during the course of the HFD feeding in hair, instead of plasma, since hair cutting causes minimal stress to mice compared to blood retrieval (Fig. 4A). Notably, corticosteroid levels in newly grown hair (2 to 3 mm above the skin) reflect the cumulative corticosteroid production over a period of 3 to 4 weeks (*29*, *30*). Already at 3 weeks after SC-26196 treatment start, levels of 11-dehydrocorticosterone in newly grown hair were reduced in HFD mice receiving the inhibitor compared to control mice (fig. S3A). 11-Dehydrocorticosterone is the inactive form of corticosterone, which is converted to the latter by 11β-HSD1 in tissues (*31*). Hence, reduced availability of 11-dehydrocorticosterone can lead to lower (active) corticosterone levels in tissues (*31*). At 6 weeks after SC-26196 treatment start, both 11-dehydrocorticosterone and corticosterone levels were reduced in the hair of mice receiving the FADS2 inhibitor compared to control mice (Fig. 4B). Lower corticosteroid production associated with reduced free PUFA, including ARA, levels in the adrenal glands of SC-26196 treated mice (Fig. 4C). Glucose tolerance was not affected by treatment of mice with SC-26196 (fig. S3B). These findings demonstrate that FADS2 inhibition in established obesity can alter the PUFA content of the adrenal glands and down-regulate adrenocortical steroidogenesis.

### Dietary supplementation with icosapent ethyl reduces corticoid levels in obese mice

**Fig. 5.**
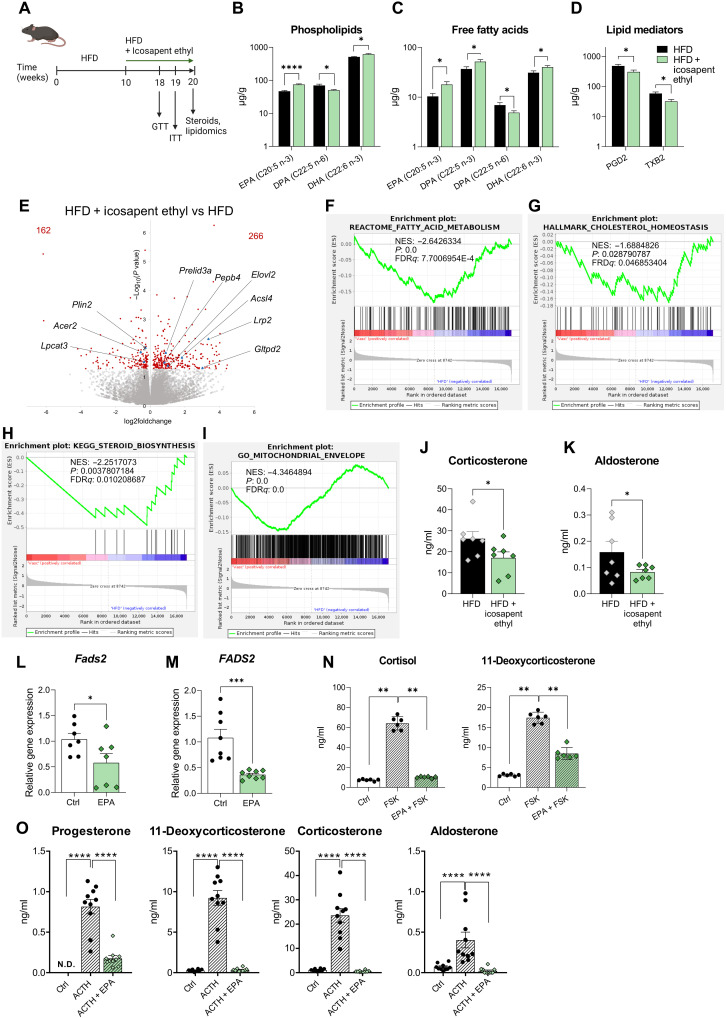
Dietary supplementation with icosapent ethyl reduces corticoid levels in obese mice. (**A**) Schematic representation of the experimental setup. (**B** to **D**) Acyl chains of phospholipids (B), FFA (C), and lipid mediators (D) were measured by HPLC-MS in the adrenal glands of mice fed a HFD with or without icosapent ethyl (*n* = 7 mice per group). (**E**) Volcano plot based on RNA-seq data in the adrenal cortex of mice, which received HFD supplemented with icosapent ethyl versus mice fed a HFD without icosapent ethyl (*n* = 4 mice per group). (**F** to **I**) GSEA analysis for genes related to fatty acid metabolism, cholesterol homeostasis, steroid biosynthesis, and mitochondrial envelope formation (*n* = 4 mice per group). (**J** and **K**) Corticosterone and aldosterone plasma levels in mice, which received HFD without and with icosapent ethyl, measured by LC-MS/MS (*n* = 7 mice per group). (**L** and **M**) Primary CD31^−^CD45^−^ adrenocortical cells (L) and NCI-H295R cells (M) were treated with 66 μM EPA for 18 or 48 hours, respectively, and *Fads2* expression was determined by qPCR using 18*S* expression as a housekeeping gene [*n* = 7 in (L) and *n* = 8 in (M)]. (**N**) NCI-H295R cells were treated for 48 hours with 66 μM EPA and stimulated or not with forskolin (FSK;1 μM) in the last 24 hours. Steroids were measured in the culture supernatant by LC-MS/MS (*n* = 6). (**O**) Primary adrenocortical cells were treated for 18 hours with 66 μM EPA and stimulated or not with ACTH (10 ng/ml). Steroids were measured in the culture supernatant by LC-MS/MS (*n* = 10). Data in (B) to (D) and (L) and (M) are presented as mean ± SEM, data in (N) and (O) as mean ± SD. *P < 0.05; **P < 0.01; ***P < 0.001; ****P < 0.0001. N.D., not determined.

To further elaborate on the requirement of FADS2-dependent PUFA synthesis for adrenocortical steroidogenesis, we asked whether enhanced activation of the FADS2-ARA axis can be regulated by dietary interventions. In general, increased intake or elevated blood levels of omega-3 lipids are associated with health benefits, while an omega-6/omega-3 imbalance is a risk factor for chronic disease (*32*, *33*). Icosapent ethyl, a highly purified and stable EPA ethyl ester, is approved as an adjunct to diet to reduce triglyceride, non–high-density lipoprotein (HDL) and apolipoprotein B levels and to lower the risk of cardiovascular events in hypertriglyceridemic patients, as shown by the recent Reduction of Cardiovascular Events with Icosapent Ethyl–Intervention Trial and other clinical studies (*34*–*36*). We therefore asked whether dietary supplementation with icosapent ethyl can lower corticosterone and aldosterone levels in mice with diet-induced obesity. To this end, WT C57BL/6J mice were fed for 10 weeks a HFD and another 10 weeks with HFD containing or not icosapent ethyl (1.125 g/kg; corresponding to approximately 180 mg/kg of animal weight) (Fig. 5A). Icosapent ethyl treatment did not alter the body weight gain, glucose, or insulin tolerance of HFD mice (fig. S4, A to C). Lipidomic analysis in the adrenal glands of these mice showed increased EPA incorporation in the phospholipids of icosapent ethyl–treated mice, as well as higher DHA and lower omega-6 DPA levels in adrenal phospholipids (Fig. 5B). Accordingly, free EPA, omega-3 DPA, and DHA were increased, and free omega-6 DPA was decreased in the adrenal glands of mice receiving icosapent ethyl (Fig. 5C). Moreover, analysis of lipid mediators (oxylipins) showed reduced prostaglandin D2 and thromboxane B2 levels in the adrenal glands of icosapent ethyl–treated mice (Fig. 5D). Hence, dietary supplementation with icosapent ethyl in obesity transforms the adrenal lipidome toward increased omega-3 lipid content and reduced levels of proinflammatory lipid mediators.

The lipidomic alterations caused by icosapent ethyl dietary supplementation were accompanied by transcriptional changes in the adrenal cortex, as shown by RNA-seq, with 266 genes being up- and 162 genes being down-regulated (Fig. 5E). Among the differentially expressed genes, several lipid metabolism–associated genes, such as *Acsl4* and *Elovl2*, were up-regulated, while others, such as *Plin2* and lysophosphatidylcholine acyltransferase 3 (*Lpcat3*), were down-regulated (Fig. 5E). GSEA analysis showed negative enrichment of genes related to fatty acid metabolism, cholesterol homeostasis, and steroid biosynthesis in the adrenal cortex of icosapent ethyl–treated mice (Fig. 5, F to H), pathways which were up-regulated in the adrenal cortex of HFD compared to LFD mice (Fig. 2, J to L). In addition, genes related to mitochondrial envelope formation, respiratory electron transport, oxidative phosphorylation, and TCA cycle were negatively enriched in the adrenal cortex of icosapent ethyl–receiving mice (Fig. 5I and fig. S4, D to F), again standing in stark contrast to the up-regulation of these pathways in the adrenal glands of HFD versus LFD mice (Fig. 2M and fig. S2, M to P). Icosapent ethyl dietary supplementation reduced corticosterone and aldosterone plasma levels in obese mice (Fig. 5, J and K) without altering ACTH plasma levels (fig. S4G). These data collectively demonstrate that dietary administration of icosapent ethyl in obesity reduces adrenocortical steroidogenesis in an HPA axis–independent fashion. Instead, it is associated with lipidomic and transcriptional reprograming of the adrenal cortex, suggesting an intra-adrenal regulation of corticosteroid production. In support of these findings, EPA treatment down-regulated *FADS2* (Fig. 5, L and M) and reduced steroidogenesis in forskolin-stimulated NCI-H295R cells (Fig. 5N) and ACTH-stimulated primary adrenocortical cells (Fig. 5O), further substantiating that FADS2 determines adrenocortical hormone production.

### FADS2 deficiency perturbs adrenal gland function

**Fig. 6.**
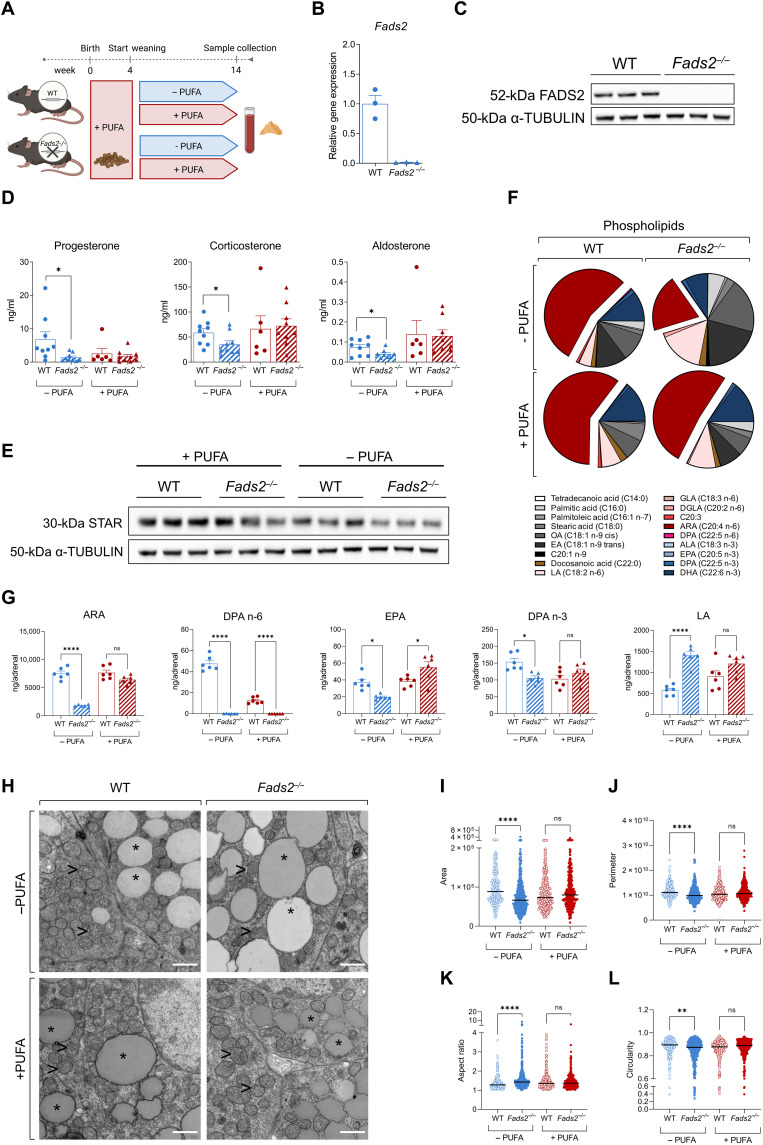
FADS2 deficiency perturbs adrenal gland function. (**A**) Scheme demonstrating the experimental setup. (**B** and **C**) FADS2 deletion efficiency in *Fads2^−/−^* mice. FADS2 expression was determined in adrenal glands of WT and *Fads2^−/−^* mice by qPCR using *Tbp* expression as a housekeeping gene (B) and Western blot using α-Tubulin as a loading control (C) (*n* = 3 mice per group). (**D**) Progesterone, corticosterone, and aldosterone levels in the plasma of WT and *Fads2^−/−^* mice fed a low-PUFA or PUFA-rich diet (*n* = 6 to 9 mice per group). (**E**) Western blot for StAR in adrenal glands of WT and *Fads2^−/−^* mice fed a low-PUFA or PUFA-rich diet. α-Tubulin was used as a loading control (*n* = 3 mice per group). (**F** and **G**) Acyl chain composition of phospholipids in the adrenal gland of WT and *Fads2^−/−^* mice fed a low-PUFA or PUFA-rich diet (*n* = 6 mice per group). Results are presented as % of all phospholipidic acyl chains (F) and as ng per adrenal gland (G). OA, oleic acid; EA, elaidic acid. (**H**) Representative electron microscopy images of adrenocortical cells; adrenal glands of two WT and two *Fads2^−/−^* mice fed a low-PUFA and 1 WT and 1 *Fads2^−/−^* mice fed a PUFA-rich diet were imaged (magnification 6,800x). Scale bars, 1 μM. Asterisks (*) depict lipid droplets, and arrowheads (>) depict mitochondria. (**I** to **L**) Quantification of mitochondrial area (I), perimeter (J), aspect ratio (K), and circularity (L) in WT and *Fads2^−/−^* mice fed a low-PUFA or PUFA-rich diet (in total, 198 to 429 mitochondria were quantified in two WT and two *Fads2^−/−^* mice fed a low-PUFA and one WT and one *Fads2^−/−^* mice fed a PUFA-rich diet). Data in (B), (D), and (G) are shown as means ± SEM. *P < 0.05; **P < 0.01; ****P < 0.0001.

To further confirm the role of the FADS2-dependent PUFA synthesis in adrenocortical function, we used *Fads2* knockout (*Fads^−/−^*) mice, which were kept for 10 weeks under a diet with low or high PUFA concentration (Fig. 6A and table S4). FADS2 deficiency was verified in the adrenal gland at mRNA and protein level (Fig. 6, B and C). Adrenal glands from *Fads2^−/−^* mice did not show any obvious abnormalities in size or macroscopic structure compared to littermate WT mice fed a PUFA-rich or low-PUFA diet (fig. S5A). However, progesterone, corticosterone, and aldosterone plasma concentrations were reduced in *Fads2^−/−^* mice fed the low-PUFA diet but not in mice fed the PUFA-rich diet (Fig. 6D). Reduced corticosteroid production was not due to reduced ACTH levels, i.e., WT and *Fads2^−/−^* mice fed with either diet displayed similar ACTH plasma levels (fig. S5B). In accordance, StAR expression was reduced in *Fads2^−/−^* mice, which were kept under low-PUFA diet but not in mice fed the PUFA-rich diet (Fig. 6E). Expression of other steroidogenic genes (*Cyp11a1*, *3*β*-Hsd2*, *Cyp11b1*, and *Cyp11b2*) was not affected by FADS2 deficiency (fig. S5C).

As outlined above, FADS2-mediated ARA synthesis is crucial for adrenal steroidogenesis. Lipidomic analysis showed that ARA abundance in adrenal phospholipids of WT mice was not affected by feeding with the low-PUFA diet (Fig. 6, F and G), suggesting that the ARA content in adrenal phospholipids is not affected by dietary ARA uptake when FADS2 is expressed. However, in *Fads2^−/−^* mice fed the low-PUFA diet the ARA content in adrenal phospholipids was strongly reduced compared to WT mice fed the same diet but not in mice fed a PUFA-rich diet (Fig. 6, F and G, and table S5). DPA (C22:5 n-6) levels in phospholipids were also diminished in the adrenal glands of *Fads2^−/−^* mice independently of the diet (Fig. 6, F and G, and table S5). The levels of EPA and DPA (C22:5 n-3) were reduced in the adrenal glands of FADS2-deficient mice receiving a low-PUFA diet (Fig. 6, F and G), although to a lesser extent than the omega-6 fatty acids ARA and DPA (C22:5 n-6), suggesting that FADS2 may rather promote the synthesis of omega-6 fatty acids. In accordance, the PUFA precursor LA increased in the adrenal glands of *Fads2^−/−^* mice fed the low-PUFA diet presumably due to its diminished use for PUFA synthesis (Fig. 6, F and G).

Moreover, adrenocortical cells of *Fads2^−/−^* mice fed the low-PUFA diet had larger lipid droplets compared to WT mice receiving the same diet, as shown by electron microscopy (Fig. 6H and fig. S5D). This stands in accordance with the observed reduced steroid hormone production (Fig. 6D); hence, the attenuated use of CE for steroidogenesis, which are thus accumulating in cells forming larger lipid droplets. Adrenocortical cells of *Fads2^−/−^* mice fed the low-PUFA diet also displayed fewer, smaller, and less circular mitochondria (Fig. 6, H to L), indicating perturbed mitochondrial function, thus standing in agreement with impaired steroidogenesis. Together, these data further indicate that FADS2-dependent PUFA synthesis determines the lipidomic landscape, mitochondrial structure, lipid droplet load, and steroidogenesis in adrenocortical cells.

### *FADS2* expression correlates with the expression of steroidogenic genes in the human adrenal gland and is increased in aldosterone-producing adenomas

To validate the significance of our findings in the human adrenal gland, we mined the public GEPIA2 database (http://gepia2.cancer-pku.cn/#index) (*37*) for steroidogenic genes and analyzed their expression in correlation with the expression of *FADS2*. *FADS2* expression positively correlated with the expression of *STAR*, *VDAC1, CYP11A1*, Steroidogenic Factor-1 (*SF-1*), and protein kinase A (PKA) catalytic subunit A (*PRKACA*) in the human adrenal gland (fig. S6, A to E). VDAC1 interacts with StAR at the OMM, facilitating cholesterol transport into mitochondria (*38*). CYP11A1 catalyzes the side-chain cleavage reaction, which is the first step of steroidogenesis generating pregnenolone (*39*). *PRKACA* mediates PKA activation, which triggers steroidogenesis, and is involved in adrenocortical pathologies with ACTH-independent hypercortisolism, such as adrenocortical adenomas, carcinomas, or bilateral adrenocortical hyperplasias (*40*). *SF-1* is the major regulator of steroidogenesis-related genes (*41*). The correlation of the expression of *FADS2* with the expression of genes that play a critical role in the initiation of steroidogenesis in the human adrenal gland underscores the relevance of our findings. On the basis of GEPIA2 data, *FADS2* also positively correlates with *SF-1* and *PRKACA* expression in adrenocortical carcinoma (fig. S6, F and G).

**Fig. 7.**
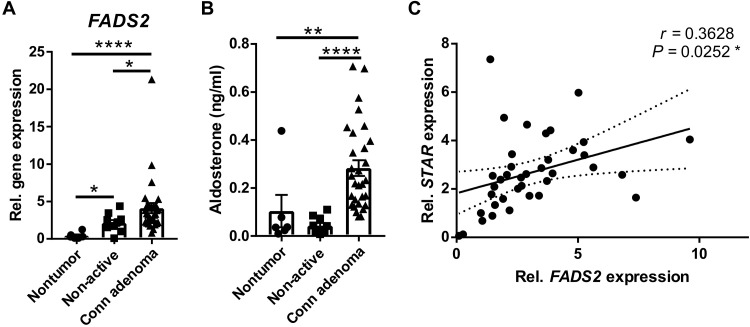
*FADS2* expression is increased in aldosterone-producing adenomas. (**A**) *FADS2* expression was determined in nontumorous adrenocortical tissue from pheochromocytoma patients (*n* = 6 patients), non-active adenomas (*n* = 10 patients), and aldosterone-producing adenomas (Conn adenomas) (*n* = 30 patients) by qPCR using 18*S* as a housekeeping gene. (**B**) Pre-operative aldosterone plasma levels in the same patients as in (A) were measured by LC-MS/MS. (**C**) Correlation of *FADS2* with *STAR* expression in aldosterone- and cortisol-producing adrenocortical adenomas from 30 and 7 patients, respectively. *FADS2* and *STAR* expression was determined by qPCR using 18*S* as a housekeeping gene. Data in (A) and (B) are presented as mean ± SEM. *P < 0.05; **P < 0.01; ****P < 0.0001

Along this line, we investigated the changes in *FADS2* expression in human adrenal tumors. *FADS2* expression was analyzed in nontumorous adrenocortical tissue, nonfunctioning adrenocortical adenomas (hormonally inactive), and aldosterone-producing adenomas (Conn adenomas) and was found to be significantly higher in aldosterone-producing adenomas compared to both, nontumorous adrenocortical tissue and nonfunctioning adenomas (Fig. 7A). Notably, *FADS2* expression was also elevated in hormonally inactive tumors compared to nontumorous adrenocortical tissue (Fig. 7A), suggesting a potential role of FADS2 in adrenocortical tumorigenesis. Increased *FADS2* expression in Conn adenomas was associated with enhanced pre-operative aldosterone levels in these patients compared to patients of the two other groups (Fig. 7B). In addition, *FADS2* expression positively correlated with *STAR* expression in aldosterone- and cortisol-producing adrenocortical adenomas (Fig. 7C). These data collectively support that FADS2 is not only required for steroidogenesis in the human adrenal gland but may also promote increased steroid production in adrenocortical adenomas.

## DISCUSSION

Chronically elevated cortisol levels can have deleterious consequences, including disturbed lipid and glucose metabolism, immunodeficiency, and neurological disorders (*3*, *22*). Increased aldosterone levels promote hypertension and increase the risk of cardiovascular disease (*1*). Moderately elevated corticoid levels in obese subjects contribute to complications such as insulin resistance and hypertension and can thus substantially affect life quality but are rarely treated (*4*, *7*, *8*). The mechanisms underlying derailed adrenocortical hormone production in obesity are incompletely understood. Here, we investigated to which extent the lipidomic landscape of the adrenal gland affects its steroidogenic function. We analyzed the adrenal lipidome of lean and obese mice and found that a high content of phospholipids in longer and more unsaturated lipids is associated with increased steroidogenesis in obese mice. ARA is a particularly abundant acyl chain in adrenal phospholipids and is increased with obesity. We identified FADS2, the rate-limiting enzyme for PUFA synthesis, as a key determinant for the adrenal lipidome and steroidogenic function. FADS2 is highly expressed in the adrenal gland compared to other tissues and increases in the adrenal gland of obese animals. Moreover, we propose that not only adrenocortical FADS2-mediated ARA synthesis but also medulla-derived ARA may affect adrenocortical steroidogenesis.

The importance of the FADS2-ARA axis for steroidogenesis was validated in *Fads2^−/−^* mice, which displayed lower corticosterone and aldosterone plasma levels compared to WT littermates when receiving for 10 weeks a low-PUFA diet, while corticoid levels were restored by a PUFA-rich diet. In accordance, adrenocortical cells in *Fads2^−/−^* mice fed a low-PUFA diet had larger lipid droplets and disturbed mitochondria. Along the same line, in vitro treatment of adrenocortical cells with SC-26196, a FADS2 inhibitor, potently reduced the abundance of PUFA, including ARA acyl chains of mitochondrial phospholipids, mainly PC and PE. These are the most abundant phospholipids in all cellular membranes including mitochondrial membranes (*14*, *15*) and important stores of ARA residues (*42*–*44*). FADS2 inhibition also reduced the mitochondrial membrane potential, oxygen consumption, and mitochondrial cholesterol uptake and diminished steroidogenesis, which was partially reversed by ARA supplementation. ARA was previously reported to be required for steroidogenesis and high amounts of arachidonic cholesterylesters are found in rat adrenal glands (*27*, *45*). However, our study suggests that the phospholipidomic landscape and the ARA content of mitochondria specifically determines steroidogenesis. On the basis of our findings, we hypothesize that the PUFA content of phospholipids assembling the mitochondrial membrane is critical for proper mitochondrial membrane potential and bioenergetics, which is pivotal for cholesterol transport into mitochondria and steroidogenesis (*10*, *16*–*19*).

Abundance of long-chain PUFA in phospholipids constructing phospholipid layers increases membrane fluidity, in contrast to saturated fatty acids, which make membranes more rigid (*46*, *47*). In turn, membrane fluidity can determine enzymatic activity and secretory functions (*46*–*49*). Steroid hormones are rapidly synthesized from cholesterol through a cascade of enzymatic reactions taking place in the mitochondria and the ER (*10*). The activity of the enzymes involved in steroid biosynthesis depends on the membrane fluidity of these organelles (*50*, *51*). Along this line, our data suggest that FADS2 providing PUFA residues may increase membrane fluidity in adrenocortical cells thereby promoting steroidogenesis. In agreement with this hypothesis, FADS2-deficient female and male mice are sterile due to changes in the membrane fluidity of gonadal cells encompassing less PUFA acyl groups (*11*, *12*). Fertility of these mice is restored by dietary supplementation with ARA, EPA, and DHA (*11*, *52*). On the other hand, FADS2 deficiency was reported to lead to a noncanonical conversion of linoleate to eicosatrienoic acid (20:3 n-6), which substitutes ARA in phospholipids thereby critically changing the structure and function of ER and Golgi membranes (*53*). However, the precise mechanisms linking the mitochondrial lipidome with steroidogenesis in the adrenal gland remain to be elucidated.

Having found that the adrenal lipidome is integral to the regulation of steroidogenesis, we next asked whether it can be modulated in established obesity by FADS2 inhibition. Along this line, we demonstrate that administration of SC-26196 through the food reduced the PUFA content of the adrenal gland and down-regulated corticosteroid production in obese mice. We also asked whether the adrenal lipidome and corticoid production can be modulated by other dietary interventions. To this end, EPA was selected as a candidate dietary component. EPA together with DHA, i.e., the two main fish oil components used for food supplementation, was previously shown to reduce corticosterone levels and anxiety in rats subjected to restrained stress test (*54*). EPA and DHA supplementation also reduced cortisol saliva levels and perceived stress in abstinent alcoholics (*55*). In addition, EPA decreased serum cortisol levels in patients with major depression disorder (*56*). Hence, the concept of omega-3 fatty acid–mediated regulation of glucocorticoid levels is not new, but the underlying mechanisms remained little understood. Here, we fed mice with HFD for 10 weeks and another 10 weeks with HFD containing or not icosapent ethyl, a highly purified EPA ethyl ester (*36*). We chose to administrate icosapent ethyl instead of EPA or EPA with DHA, based on the findings of large clinical studies, which showed that icosapent ethyl alone lowered the risk of cardiovascular events, non-HDL cholesterol, and triglycerides in patients with established cardiovascular disease, hypertriglyceridemia, or diabetes (*34*–*36*). We demonstrate that icosapent ethyl food supplementation increased the omega-3 fatty acid content of the adrenal lipidome, instigated transcriptional changes in the adrenal cortex opposite to the ones evoked by HFD, which were associated to lipid metabolism, mitochondrial function and steroid biosynthesis, and reduced corticosterone and aldosterone plasma levels. Accordingly, EPA treatment of cultured adrenocortical cells reduced FADS2 expression and diminished forskolin-stimulated corticoid production, indicating that icosapent ethyl dietary supplementation may directly regulate the steroidogenic function of adrenocortical cells. In agreement with these results, EPA was previously shown to down-regulate *FADS2* expression in THP-1 cells (*57*), 3T3-L1 adipocytes (*58*), and HepG2 hepatocytes (*59*). Hence, icosapent ethyl is likely to influence the lipidome of not only the adrenal gland but also several other tissues.

Last, to substantiate the role of FADS2 in the human adrenal gland, we analyzed its expression in adrenal nontumorous and tumorous tissue. We demonstrate that *FADS2* expression is higher in aldosterone-producing adenomas compared to nonfunctioning adenomas or nontumorous adrenocortical tissue and correlates with the expression of *STAR* in aldosterone- and cortisol-producing adenomas.

These data collectively demonstrate that FADS2 is a major regulator of steroidogenesis in the adrenal gland due to its key role in shaping the lipidomic landscape of adrenocortical cells. Moreover, our findings indicate that the adrenal lipidome can be modulated by icosapent ethyl dietary supplementation, which consequently influences corticoid production, thereby endorsing the evaluation of icosapent ethyl dietary supplementation as a means of regulation of cortisol and aldosterone levels in obese subjects. Last, our data indicate a clear correlation between FADS2 expression and steroidogenic capacity in the human adrenal gland and suggest that the role of FADS2–mediated lipidomic changes in adrenocortical tumorigenesis merits further investigation.

## MATERIALS AND METHODS

### Mice and in vivo experiments

The *Fads2^−/−^* mice were developed by Stroud *et al.* (*12*). The first 4 postnatal weeks female WT and *Fads2^−/−^* littermate mice were kept with their mothers and fed a PUFA-rich diet. After weaning at 4 weeks, they were kept for another 10 weeks on a PUFA-rich or low-PUFA diet. Composition of the diets is shown in table S4.

Six-weeks-old male WT C57BL/6J mice were fed for 20 weeks a LFD (D12450B) or a HFD (D12492) both from Research Diets, NJ, USA. In some experiments, WT C57BL/6J mice were fed for 8 weeks a HFD and for another 8 weeks a HFD supplemented with SC-26196 (Biorbyt Ltd.). SC-26196 was mixed in the HFD at a concentration of 625 mg/kg of diet corresponding to approximately 100 mg/kg of animal weight. In other experiments, WT mice were fed for 10 weeks a HFD and for another 10 weeks a HFD supplemented with icosapent ethyl (Vascepa). The content of Vascepa capsules was mixed by Research Diets with the diet at a concentration of 1.125 g/kg icosapent ethyl (corresponding to approximately 180 mg/kg of animal weight). Mice were weighed every week. Glucose and insulin tolerance tests were performed as previously described (*20*, *21*). Three to 5 mg of hair (2 to 3 mm close to the skin on the back of the mice) was cut while gently holding the mice without anesthesia.

At the end of the experiment, blood was collected retroorbitally in EDTA tubes, the mice were sacrificed by cervical dislocation, and organs were excised. Tissues were immediately snap-frozen or processed for cell isolation. For electron microscopy, mice were anesthetized with isoflurane and systemically perfused with 4% paraformaldehyde (PFA), 0.1% glutaraldehyde (GA), 0.1 M sodium phosphate buffer, or phosphate-buffered saline (PBS) (pH 7.4). Experiments were approved by the Landesdirektion Sachsen, Germany and the institutional animal care and use committee of Azabu University.

### Human samples

The study included retrospective data and biosamples from 53 patients who underwent surgery because of adrenal tumors, 10 patients with nonfunctional adenomas, 30 patients with aldosterone-producing adenomas (Conn adenomas), 7 with cortisol-producing adenomas, and 6 patients with pheochromocytoma. Patients were enrolled in the ENSAT and the NeoExNet registry studies and the prospective clinical trials, Prospheo/Muppet (https://muppet.eu/) and PMT-trial (pmt-study.pressor.org) in three tertiary centers in Germany: University Hospital Carl Gustav Carus, TU Dresden, University Hospital Wuerzburg, and University Hospital Munich, Ludwig-Maximilians-Universität München. The studies were approved by the Ethics Committees at the Medical Faculty of each University. All patients provided written informed consent. Measurements of steroids in plasma were performed using LC-MS/MS (*60*). Potential contamination with medulla tissue was excluded in all samples by examination of tyrosine hydroxylase (*TH*) and phenylethanolamine *N*-methyltransferase (*PNMT*) expression by quantitative polymerase chain reaction (qPCR). Among the limitations of the study design is that possible influences from antihypertensive medication interfering with the renin-angiotensin-aldosteron system were not considered in the analysis.

### Sorting of adrenocortical cell populations

Adrenal glands were isolated and cleaned from the surrounding fat tissue, and the adrenal cortex was separated from the medulla under a dissecting microscope. The cortex was collected in a digestion buffer, consisting of collagenase I and bovine serum albumin (BSA; both at 1.6 mg/ml concentration; Sigma-Aldrich) dissolved in PBS, and digested for 25 min at 37°C while shaking at 900 rpm. Dissociated cells were passed through a 22-G needle and then a 100-μm cell strainer and centrifuged at 300*g* for 5 min at 4°C. Cells were then washed in MACS buffer (0.5% BSA and 2 mM EDTA in PBS), and endothelial cells (CD31^+^) and leukocytes (CD45^+^) were sequentially positively selected using CD31 and CD45 MicroBeads (Miltenyi Biotec), respectively, according to manufacturer’s instructions. Briefly, pelleted cells were resuspended in 190-μl MACS buffer, mixed with 10-μl CD31 MicroBeads, incubated for 15 min at 4°C, washed with 2-ml MACS buffer, and centrifuged at 300*g* for 10 min. The cell pellet was resuspended in 500-μl MACS buffer and applied onto MS Column placed on a MACS Separator. The columns were washed and the flow-through was collected. CD31^+^ cells were positively sorted. The flow-through was centrifuged at 300*g* for 5 min, and the pelleted cells were subjected to the same procedure using CD45 MicroBeads. The flow-through containing CD31^−^CD45^−^ adrenocortical cells was centrifuged at 300*g* for 5 min, and the pelleted cells were collected. CD45^+^ cells were positively sorted. Collected cell populations were kept for transcriptional analysis (*61*).

### Cell and explant culture and treatments

Adrenal glands or adrenal corteces were isolated from WT C57BL/6J mice, cleaned from the surrounding fat tissue, and incubated in collagenase buffer [collagenase type I (1.6 mg/ml) and BSA (1.6 mg/ml)] for 45 min at 37°C while shaking at 900 rpm. Dissociated cells were passed through 22-G needle and a 100-μm cell strainer and centrifuged at 300*g* for 5 min at 4°C. Cells of a pool from two adrenals of each mouse were distributed in four 0.2% gelatin-precoated wells of a 96-well plate in DMEM/F12 supplemented with 1% fetal bovine serum, penicillin (50 U/ml), and streptomycin (50 μg/ml). Two hours after seeding, 10 μM SC-26196 (Tocris) or the same amount of carrier [dimethyl sulfoxide (DMSO)] was added without changing the medium. In some experiments, medium was additionally supplemented with 150 μM ARA (Sigma-Aldrich) or same amount of solvent (endotoxin-free water). When induction of steroidogenesis was required, the medium was changed 18 hours after treatment, and the cells were incubated with or without ACTH (10 ng/ml) for 60 min in the presence of either 10 μM SC-26196 or DMSO. Supernatants were kept for steroid hormone measurement, and cell lysates were collected for transcriptional analysis. In some experiments, primary CD31^−^CD45^−^ adrenocortical cells were treated or not for 18 hours with 66 μM EPA (Sigma-Aldrich) followed or not by ACTH stimulation (*61*).

Medullas were isolated from WT C57BL/6J mice and transferred into DMEM/F12 medium. Six hours later, the medulla explant supernatants were collected and applied onto adrenocortical cell cultures. These were previously treated for 6 hours with SC-26196 (10 μM) or DMSO. SC-26196 or DMSO was re-added, and adrenocortical cells were incubated for another 18 hours until supernatants were collected for steroid measurement. In other experiments, medulla explants were treated for 6 hours with SC-26196 (10 μM) or DMSO, washed thoroughly, and left another 18 hours in culture. Their supernatant was then applied on adrenocortical cell cultures, and 8 hours later, supernatants of the latter were collected and analyzed by LC-MS/MS.

NCI-H295R cells (obtained from American Type Culture Collection catalog no. CRL-2128) were maintained in DMEM/F12 medium supplemented with 2.5% Nu serum type I (Corning), 1% insulin transferrin selenium (Gibco), penicillin (50 U/ml), and streptomycin (50 μg/ml). They were treated for 18 hours with 10 μM SC-26196 or DMSO. In some experiments, NCI-H295R cells were treated or not for 48 hours 66 μM EPA, and in the last 24 hours, they were stimulated or not with forskolin (10 μM; Sigma-Aldrich) (*61*).

### Cell fractionations

Cells were trypsinized and pelleted by centrifugation (500*g*, 5 min, 4°C). The cells were gently resuspended in homogenization buffer [200 mM mannitol, 50 mM sucrose, 10 mM KCl, 1 mM EDTA, 25 mM Hepes (pH 7.4), 1 μM aprotinin, 10 μM leupeptin, and 20 μM phenylmethylsulfonyl fluoride] and homogenized on ice with 20 strokes with a Dounce homogenizer. The samples were then centrifuged (800*g*, 10 min, 4°C) to pellet the nuclei. The supernatant was mixed with iodixanol solution (Optiprep, Sigma-Aldrich) to a final concentration of 20%. The samples were placed at the bottom of a tube and overlaid with 500 μl of iodixanol solutions (18, 16, 14, 12, 10, and 5%) and lastly with 500 μl of Hepes-buffered saline (HBS) [50 mM Hepes and 150 mM NaCl (pH 7.4)]. The samples were centrifuged in a Beckman SW60Ti swing-out rotor (45,000 rpm, 5 hours, 4°C). After centrifugation, 15 280-μl aliquots were collected from top of the tubes.

Fractions 10 to 12 (SDHB-positive) and 4 to 5 (HSL-positive) were considered as mitochondrial and lipid droplet fractions, respectively. Protein concentration was measured in the mitochondrial and lipid droplet pools with the BCA Protein Assay Kit (Thermo Fisher Scientific), and fractions were analyzed for cholesterol and CE concentrations.

### Lipidomic analysis of isolated mitochondria

Fractions containing mitochondria were combined and extracted with three volumes of ice-cold chloroform/methanol [10:1, v/v, both containing 0.1% butylated hydroxytoluene (Merck, ≥99%)]. Samples were incubated on ice for 15 min, before the organic phase was collected after centrifugation (5 min, 5,000*g*). The aqueous phase was re-extracted with two volumes chloroform/methanol as described above. Combined organic phases were dried in vacuo. Methanol (ULC-MS grade, Biosolve B.V.) and chloroform (EMSURE ACS, ISO, Reag. Ph Eur; Supleco) were of highest purity.

Lipid extracts were dissolved in 50 μl of 2-propanol (ULC/MS-CC/SFC grade, >99.95%; Biosolve B.V.) and analyzed by LC-MS/MS. Lipids (2.5 μl loaded in positive mode, 5 μl in negative mode) were separated by reverse phase chromatography (Accucore C30 column; 150 mm by 2.1 mm, 2.6 μM, 150 Å; Thermo Fisher Scientific) using a Vanquish Horizon ultrahigh-performance liquid chromatography (UHPLC) system (Thermo Fisher Scientific) coupled on-line to a Q Exactive Plus Hybrid Quadrupole Orbitrap mass spectrometer (Thermo Fisher Scientific) equipped with a heated electrospray ionization (HESI) source. Column was operated at 50°C and a flow rate of 0.3 ml/min. Chromatographic gradient included following steps: 0 to 20 min, 10 to 86% B; 20 to 22 min, 86 to 95% B; 22 to 26 min, 95% isocratic; 26 to 26.1 min, 95 to 10% B, followed by 5 min re-equilibration at 10% B. Eluent A consisted of acetonitrile/water (50:50, v/v, both ULC/MS-CC/SFC grade, Biosolve B.V.) and Eluent B of 2-propanol/acetonitrile/water (85:10:5, v/v/v), both containing 5 mM ammonium formate (MS grade, Sigma-Aldrich) and 0.1% formic acid (ULC/MS-CC/SFC grade, Biosolve B.V.). HESI parameters are as follows: sheath gas, 40 arbitrary units; auxiliary gas, 10 liters/min; sweep gas, 1 liter/min; spray voltage, 3.5 kV (positive ion mode) and 2.5 kV (negative ion mode); ion transfer temperature, 300°C; S-lens radio frequency (RF) level, 35%; aux gas heater temperature, 370°C. Data were acquired using data-dependent acquisition: full scan resolution 140,000 at mass/charge ratio (*m/z*) 200, AGC target 1 × 10^6^, maximum IT 100 ms, scan range *m/z* 350 to 1200; data-dependent MS/MS: resolution 17,500 at *m/z* 200, AGC target 1 × 10^5^, maximum IT 60 ms, loop count 15, isolation window 1.2 *m/z*, stepped normalized collision energies of 10, 20, and 30%, AGC target of 2 × 10^2^, dynamic exclusion 10 s, isotope exclusion on, default charge state 1 (*62*).

Lipid identification and relative quantification was performed using Lipostar2 (*63*). Briefly, supersample filter considering only lipids with isotopic pattern and with an MS/MS spectrum were kept. Features were searched against the LIPID MAPS structural database (downloaded November 2021) and proposed identifications obtained by automatic approval considering three and four stars. Data were normalized using the Sum/Average normalization (area sum). Statistical analysis was performed in MetaboAnalyst 5.0 (sample normalization by median, data log transformed, pareto scaled) (*64*).

### Product ion scan for cholesterol and cholesteryl esters

For quantification of cholesterol and cholesteryl esters, equal volumes of mitochondria fractions (180 μl) were combined, cholesterol-2,3,4-^13^C (5 nmol; Sigma-Aldrich) and SPLASHLipidomix (3 μl; Avanti Polar Lipids Inc) spiked, and samples were incubated for 15 min on ice. Lipids were extracted as described above.

Lipid extracts were reconstituted in 55 μl of 2-propanol, and 10 μl was loaded onto a Accucore C30 column (150 mm by 2.1 mm, 2.6 μM, 150 Å; Thermo Fisher Scientific) installed on a Vanquish Horizon UHPLC (Thermo Fisher Scientific) coupled on-line to a Orbitrap Exploris 240 (Thermo Fisher Scientific) mass spectrometer equipped with a HESI source. Lipids were separated at a flow rate of 0.3 ml/min and a column temperature of 50°C by a gradient starting from 10% B (0 to 20 min 10 to 80% B, 20 to 37 min 80 to 95% B, 37 to 41 min 95 to 100% B, 41 to 49 min 100% B, and 49.1 to 57 min 10% B). The column was re-equilibrated for 7.9 min at 10% eluent B. Eluent A consisted of acetonitrile/water (50:50, v/v, both ULC/MS-CC/SFC grade Biosolve B.V.) and eluent B of 2-propanol/acetonitrile/water (85:10:5, v/v/v), both containing 5 mM Ammonium formate (MS grade, Sigma-Aldrich) and 0.1% formic acid (ULC/MS-CC/SFC grade, Biosolve B.V.).

For cholesterol and CE, a scheduled product ion scan method with the following parameters was used: spray voltage, 3500 V; sheath gas, 40 arbitrary units; aux gas, 10 arbitrary units; sweep gas, 1 arbitrary units; vaporizer, 370°C; ion transfer tube, 300°C; polarity, positive; default charge state, 1; isolation window, 1 *m/z*; resolution at *m/z* 200 to 45,000; HCD collision energy (%), 17, 27, 37; fixed first mass, 70 *m/z*; AGC target, standard; maximum injection time, auto; EASY-IC, start run. Quantification was performed in Skyline (version 22.2.0.255, MacCoss Lab, University of Washington) using cholesten ion (*m/z* 369.35) as a quantifier (*65*). CE was quantified relative to the 18:1-d7-cholesteryl ester present in the SPLASHLipidomix mix. Signal of cholesterol-2,3,4-^13^C was used to quantify endogenous cholesterol.

### Shotgun lipidomics and analysis

Shotgun lipidomics were performed as previously described (*61*). Adrenal glands were homogenized in ammonium-bicarbonate buffer (150 mM ammonium bicarbonate, pH 7) with TissueLyser (Qiagen). Protein content was assessed using the BCA Protein Assay Kit (Thermo Fisher Scientific). Equivalents of 20 μg of protein were taken for MS analysis. MS-based lipid analysis was performed as described (*24*). Lipids were extracted using a two-step chloroform (Sigma-Aldrich)/methanol (Thermo Fisher Scientific) procedure (*66*). Samples were spiked with internal lipid standard mixture containing: CL 16:1/15:0/15:0/15:0, Cer 18:1;2/17:0, HexCer 18:1;2/12:0, LPA 17:0, LPC 12:0, LPE 17:1, LPG 17:1, LPI 17:1, LPS 17:1, PA 17:0/17:0, PC 17:0/17:0, PE 17:0/17:0, PG 17:0/17:0, PI 16:0/16:0, PS 17:0/17:0, CE 20:0, SM 18:1;2/12:0;0, cholesterol D6 (all Avanti Polar Lipids), TAG 17:0/17:0/17:0, and DAG 17:0/17:0 (both Larodan Fine Chemicals). Synthetic lipid standards were purchased from Avanti Polar Lipids, Larodan Fine Chemicals, and Sigma-Aldrich, and all chemicals were analytical grade. After extraction, the organic phase was transferred to an infusion plate and dried in a speed vacuum concentrator (Martin Christ). The dry extract from the first step was resuspended in 7.5 mM ammonium acetate (Sigma-Aldrich) in chloroform/methanol/propan-2-ol (Thermo Fisher Scientific) (1:2:4 V:V:V) and the second-step dry extract in 33% ethanol of methylamine (Sigma-Aldrich)/chloroform/methanol (0.003:5:1 V:V:V) solution. All liquid handling steps were performed using Hamilton Robotics STARlet robotic platform with the Anti Droplet Control feature for organic solvents pipetting.

Samples were analyzed by direct infusion on a QExactive mass spectrometer (Thermo Fisher Scientific) equipped with a TriVersa NanoMate ion source (Advion Biosciences, Ithaca, NY). Samples were analyzed in both positive and negative ion modes with a resolution of *R*_(*m/z*=200)_ = 28,0000 for MS and *R*_(*m/z*=200)_ = 17,500 for MS/MS experiments, in a single acquisition. MS/MS was triggered by an inclusion list encompassing corresponding MS mass ranges scanned in 1-Da increments (*67*). Both MS and MS/MS data were combined to monitor CE, DAG, and TAG ions as ammonium adducts; PC, PC O-, as acetate adducts; and CL, PA, PE, PE O-, PG, PI, and PS as deprotonated anions. MS only was used to monitor LPA, LPE, LPE O-, LPI, and LPS as deprotonated anions; Cer, HexCer, SM, LPC, and LPC O- as acetate adduct and cholesterol as ammonium adduct of an acetylated derivative (*68*).

Data were analyzed with an in-house developed lipid identification software based on LipidXplorer (*69*, *70*). Data postprocessing and normalization were performed using an in-house developed data management system. Only lipid identifications with a signal-to-noise ratio > 5 and a signal intensity fivefold higher than in corresponding blank samples were considered for further data analysis. The dataset was filtered by applying an occupational threshold of 50%, meaning that lipids that were present in less than 50% of the samples in a group were set to zero in that group. Dataset was log_2_-scaled before PCA. The descriptive analysis was performed on the mol %–transformed dataset, i.e., picomol quantities were divided by the sum of the lipids detected in the respective sample and multiplied by 100. Total carbon chain length and double bonds plots result from grouping together all the lipids that present the same number of carbon atoms (total length) or the same number of double bonds (degree of unsaturation) and calculating the mean and standard deviation in each group of samples (LFD and HFD). To further inspect general trends in the degree of unsaturation and length of storage and membrane lipids under different perturbations, the average weighted number of double bonds (DBI) and length (LI), as previously described (*71*). Statistical tests were performed on the mol %–transformed data. First, normality was assessed by means of the Shapiro-Wilkinson test, and according to its result, either the Welch *t* test or the Wilcoxon test was used. *P* values were then adjusted following the Benjamini-Hochberg correction. Analysis was done in R (*72*). Plots were generated using ggplot2 (*73*).

### Lipid isomer and oxylipin measurement

Frozen adrenal glands were homogenized in 200 μl of PBS buffer. Lipids were extracted from adrenal lysates or supernatants in chloroform/methanol (Folch’s protocol). The extract was evaporated to dryness under stream of nitrogen at 40°C. The residue was dissolved in 100 μl of ethanol. For FFA, 50 μl were used. HPLC measurement of FFA was performed using an Agilent 1290 HPLC system with binary pump, autosampler, and column thermostat equipped with a Phenomenex Kinetex-C18 column 2.6 μm, 2.1 by 150 mm column (Phenomenex) using a solvent system of acetic acid (0.05%) and acetonitrile. All solvents and buffers in LC-MS–grade were purchased from VWR Germany. The HPLC was coupled with an Agilent 6470 triplequad mass spectrometer with electrospray ionisation source operated in negative multiple reaction monitoring. The quantification was performed with individual calibration curves using deuterated standards (Cayman Chemical).

To release fatty acids and lipid mediators from phospholipids, 50 μl lipid extract was dissolved with 500 μl of phospholipase A2 (PLA2) buffer consisting of 80 mM Hepes, 300 mM NaCl, 20 mM CaCl_2_, and BSA (2 mg/ml) in 60% glycerol (pH 7.4). This solution was spiked with 5 μl of butylhydroxytoluen and 2.2 U/26 μl of PLA2 from honey bee (Merck) and incubated 1 hour at 25°C. One hundred microliters of hydrolysate was analyzed as described above. The results were the sum of FFAs and fatty acids from phospholipids; hence, the amount of fatty acyl chains of phospholipids was calculated by subtracting the amount of FFA from phospholipidic fatty acyl chains.

For measurement of lipid mediators, 400 μl of hydrolysate was spiked with 100 pg of d4-PGE2-13 as an internal standard (Cayman Chemical). Acetonitrile (400 μl) was added for protein precipitation. After centrifugation and pH adjustment at 6.0, the obtained supernatant was added to Bond Elute Certify II columns (Agilent Technologies) for solid phase extraction. The eluate was evaporated on a heating block at 40°C under a stream of nitrogen to obtain solid residues, which were dissolved in 100 μl of methanol/water (60/40). The residues were analyzed using an Agilent 1290 HPLC system with binary pump, multisampler, and column thermostat with a Zorbax Eclipse plus C-18, 2.1 by 150 mm, 1.8-μm column using a gradient solvent system of aqueous acetic acid (0.05%) and acetonitrile/methanol 50:50. The flow rate was set at 0.3 ml/min, and the injection volume was 20 μl. The HPLC was coupled with an Agilent 6495 Triplequad mass spectrometer (Agilent Technologies) with electrospray ionisation source. Analysis was performed with multiple reaction monitoring in negative mode, at least two mass transitions for each compound.

### Steroid hormone measurement

Steroid hormones in plasma and cell culture supernatants were analyzed by LC-MS/MS as previously described (*60*, *61*). Fifty to 100 μl of plasma or cell culture supernatants were extracted by solid phase extraction using positive pressure, followed by a dry-down under gentle stream of nitrogen. Residues were reconstituted in 100 μl of the initial LC mobile phase, and 10 μl were injected for detection by the triple quadrupole mass spectrometer in multiple reaction-monitoring scan mode using positive electrospray ionization. Quantification of steroid concentrations was done by comparisons of ratios of analyte peak areas to respective peak areas of stable isotope-labeled internal standards obtained in samples to those of calibrators.

Corticosterone and 11-dehydrocorticosterone in hair were extracted with methanol as described elsewhere (*74*). Briefly, hair (3 to 5 mg) was washed by addition of 2.5 ml of isopropanol. After 3 min, the isopropanol was decanted, and hair samples were dried in a fume hood. After drying, hair samples were extracted in 1.8 ml of methanol that contained the internal standard mixtures for 18 hours at room temperature (RT). The methanolic extract was dried down, followed by reconstitution in respective mobile phase and analysis by LC-MS/MS as previously described (*60*) with minor modifications. Briefly, a QTRAP 6500+ (Sciex, Darmstadt) coupled to an Aquity i-class ultra-performance liquid chromatography system (Waters, Eschborn) was used. Chromatographic separation was achieved as described before (*60*). Corticosterone and 11-dehydrocorticosterone were detected by using the multi reaction monitoring scan mode with respective quantifier ions of 347.1->329.2 and 345.1->121.1 and qualifier ions of 347.1->90.9 and 345.1->242.3. An automatic flow injection analysis revealed optimal ion source settings of curtain gas (*40*), ion spray voltage (5500), temperature (500), gas 1 (*70*), and gas 2 (*50*). Quantification of steroid hormones was achieved by comparisons of ratios of analyte peak areas to internal standard peak areas observed in samples to those in calibrators. Measured steroid concentrations were normalized to sample weights.

### ACTH measurement

ACTH was measured in EDTA mouse plasma 4× diluted in PBS with an enzyme-linked immunosorbent assay (ELISA) kit (Abnova, KA0917) according to the manufacturer’s instructions.

### RNA sequencing

For transcriptome mapping, strand-specific paired-end sequencing libraries from total RNA were constructed using a TruSeq stranded Total RNA kit (Illumina Inc). Sequencing was performed on an Illumina HiSeq3000 (1 × 75 base pairs). Low-quality nucleotides were removed with the Illumina fastq filter, and reads were further subjected to adaptor trimming using cutadapt (*75*). Alignment of the reads to the mouse genome was done using STAR Aligner (*76*) using the parameters: “–runMode alignReads –outSAMstrandField intronMotif –outSAMtype BAM SortedByCoordinate --readFilesCommand zcat”. Mouse Genome version GRCm38 (release M12 GENCODE) was used for the alignment. The parameters: “htseq-count -f bam -s reverse -m union -a 20,” HTSeq-0.6.1p1 (*77*) were used to count the reads that map to the genes in the aligned sample files. The GTF file (gencode.vM12.annotation.gtf) used for read quantification was downloaded from Gencode (https://gencodegenes.org/mouse/release_M12.html). Gene centric differential expression analysis was performed using DESeq2_1.8.1 (*78*). The raw read counts for the genes across the samples were normalized using “rlog” command of DESeq2, and subsequently, these values were used to render a PCA plot using ggplot2_1.0.1 (*79*).

Pathway and functional analyses were performed using GSEA (*79*) and EGSEA (*80*). GSEA is a standalone software with a graphical user interface. To run GSEA, a ranked list of all the genes from DESeq2-based calculations was created usingthe −log_10_ of the *P* value. This ranked list was then queried against Molecular Signatures Database–, Kyoto Encyclopedia of Genes and Genomes (KEGG)–, Gene Ontology–, Reactome-, and Hallmark-based repositories. EGSEA is an R/Bioconductor-based command line package. For doing functional analyses using EGSEA, a differentially expressed list of genes with parameters log_2_ foldchange > 0.3 and *P* adjusted <0.05 was used. The KEGG database repository was used for performing the functional analyses.

For constructing heatmaps, the “rlog-normalized” expression values of the significantly expressed genes (*P* adjusted < 0.05) were scaled using z-transformation. The resulting matrices were visually rendered using MORPHEUS.

### Electron microscopy

After animal perfusion with PFA/GA, adrenal glands were immediately transferred into 4% PFA, 2% GA, 0.1 M sodium phosphate buffer, or PBS (pH 7.4) for 1 hour at RT and stored in 0.1% GA and 1% PFA in PBS at 4°C until processing. After washing with PBS, adrenal glands were further dissected into three pieces. They were contrasted with 1% osmium tetroxide/1.5% potassium cyanoferrate for an hour at RT. Contrast was further enhanced by incubation with 0.5% uranyl acetate overnight in the cold. Dehydration with a graded series of ethanol followed (10 min each step, three steps of pure ethanol for 20 min). Samples were gradually infiltrated and embedded in Epon resin and cured at 60°C for 48 hours. Ultrathin sections (70 nm) were cut on a Leica UC7 ultramicrotome and poststained with uranyl and lead. Samples were evaluated and images acquired on a Tecnai Biotwin T12 transmission electron microscope (Philips/FEI/Thermo Fisher Scientific) with a digital F416 charge-coupled device camera (Tietz Video and Image Processing Systems).

### Quantification of mitochondrial structural features

Mitochondrial features (area, perimeter, circularity, and aspect ratio) were analyzed in electron microscope images using the Fiji software (*81*) and the “particle analysis” plugin. Outlines of mitochondria were marked by hand using the “freehand selection” tool. At least 16 images obtained from different areas were analyzed per adrenal gland.

### Transcriptional analysis

RNA was isolated from frozen tissues with the TRI Reagent (Molecular Research Center, Inc.) after mechanical tissue disruption or from cells with the RNeasy Mini Kit (Qiagen) according to the manufacturer’s instructions. RNA obtained with TRI Reagent was subsequently extracted with Chloroform and the NucleoSpin RNA, Maxi kit (Macherey-Nagel). Reverse transcription was performed with the iScript cDNA Synthesis kit (Bio-Rad), and cDNA was analyzed by qPCR using the SsoFast Eva Green Supermix (Bio-Rad), a CFX384 real-time System C1000 Thermal Cycler (Bio-Rad) and the Bio-Rad CFX Manager 3.1 software (*61*). Primers used are listed in table S6.

### Plasmid transfection

NCI-H295R cells were transfected with a FADS2-overexpressing plasmid (Origene NM_004265) or control pCMV plasmid. Briefly, cells were plated in 24-well plates at 70% confluence, and the next day, they were transfected with 0.3 μg of DNA using Lipofectamine LTX with Plus Reagent (Thermo Fisher Scientific), according to the manufacturer’s instructions.

### Western blot analysis

Cells or tissues were lysed with 10 mM tris-HCl (pH 7.4) + 1% SDS + 1 mM sodium vanadate, cell lysates were centrifuged at 16,000*g* for 5 min at 4°C, and supernatants were collected. Total protein concentration was measured using the BCA Protein Assay Kit (Thermo Fisher Scientific) in cell lysates or cell fractions. Protein samples were prepared with 5× Reducing Laemmli buffer containing same amount of protein, denatured at 95°C for 5 min, and loaded on a 10% acrylamide gel (Invitrogen) for SDS–polyacrylamide gel electrophoresis. PageRuler Prestained Protein Ladder (Thermo Fisher Scientific) was used as a protein size ladder. The separated proteins were transferred on Amersham Protran nitrocellulose membrane (GE Healthcare Lifescience). After blocking with 5% skimmed milk in Tris-buffered saline with Tween20 (TBS-T) [0.1% Tween 20 (Sigma-Aldrich) in 1× tris-buffered saline] for 1 hour at RT, membranes were incubated overnight at 4°C with anti-FADS2 (1:2000; Invitrogen, PA5-87765), anti-StAR (1:300; Santa Cruz, SC-25806), anti-SDHB (1:1000; Sigma-Aldrich, HPA002868), anti-HSL (1:1000, Cell Signaling, #4107), anti-tubulin (1:3,000; Sigma-Aldrich, T5168) or anti-vinculin (1:3000; Cell Signaling #4650) and diluted in 5% BSA in TBS-T. After washing, membranes were incubated for 1 hour at RT with secondary antibodies: goat anti-rabbit immunoglobulin G (IgG) horseradish peroxidase (HRP)–conjugated (1:3000; Jackson ImmunoResearch) or goat anti-mouse IgG HRP-conjugated (1:3000; Jackson ImmunoResearch), diluted in 5% skimmed milk in TBS-T. The signal was detected using the Western blot Ultra-Sensitive HRP Substrate (Takara) and imaged using the Fusion FX Imaging system (PeqLab Biotechnologie).

### Immunofluorescence

Immunofluerescent stainings were performed as previously described (*82*). Adrenal glands cleaned from surrounding fat tissue were fixed in 4% PFA in PBS, washed overnight in PBS, cryopreserved in 30% sucrose (AppliChem GmbH) in PBS overnight at 4°C, embedded in Optimal Cutting Temperature (O.C.T.) compound (Tissue-Tek) and frozen at −80°C. Each adrenal gland was cut into 8-μm-thick serial sections. Before staining, adrenal sections were prewarmed at RT for 30 min. Adrenal sections were washed twice with PBS, permeabilized with 0.5% Triton X-100 in PBS for 15 min, treated with TrueBlack Lipofuscin Quencher (1:40 in 70% ethanol; Biotium) for 30 s to reduce autofluorescence, and blocked in Dako Protein Block, serum-free buffer for 1 hour at RT. Then, sections were incubated overnight at 4°C with anti-FADS2 (1:250, Invitrogen, PA5-87765), washed with PBS, and incubated for 2 hours at RT with the secondary antibody Alexa Fluor 555 donkey anti-rabbit (1:300; Thermo Fisher Scientific, #A-31572) together with 4′,6-diamidino-2-phenylindole (DAPI; 1:5000; Roche), both diluted in Dako Antibody Diluent. After washing with PBS, cryosections were mounted with Fluoromount (Sigma-Aldrich), covered with 0.17-mm cover glass, fixed with nail polish, and kept at 4°C until imaging. Adrenal cross sections were imaged as tile sections taken with an Axio Observer.Z1 (Zeiss) and a Plan-Apochromat 20× objective.

### Histology

Adrenal glands were fixed in 4% PFA and paraffin-embedded. Sections were stained with hematoxylin and eosin. Images were obtained using an Axio Observer.Z1 (Zeiss) and a Plan-Apochromat 20× objective.

### Flow cytometry

Adrenocortical cells (CD31^−^CD45^−^) were treated for 18 hours with DMSO or SC-26196 (10 μM) and were then incubated with Mitotracker Green (0.25 μM; Invitrogen), tetramethylrhodamine, ethyl ester (TMRE) (2.5 μM; Thermo Fisher Scientific), for 30 min at 37°C in FACS buffer (0.5% BSA and 2 mM EDTA in PBS) in dark. Live cells were selected by DAPI staining. FACS was performed using LSR Fortessa X20 flow cytometer, and data were analyzed with the FlowJo software.

### Seahorse analysis

Primary adrenocortical cells were plated in 0.2% gelatin-precoated XF96 cell culture microplate (Agilent) (8*10^4^ cells per well) and treated for 18 hours with DMSO or SC-26196 (10 μM). OCR was measured with a Seahorse XF96 Analyzer (Agilent Technologies). The experimental medium used was XF Base Medium supplemented with glucose (10 mM), pyruvate (1 mM), and glutamine (2 mM).

### Statistical analysis

Data were analyzed with Mann-Whitney *U* test, Students *t* test, one-way analysis of variance (ANOVA) with post hoc Tukey’s test for multiple comparisons or Pearson analysis with *P* < 0.05 set as a significance level using the GraphPrism 7 software.

## Notes

View/request a protocol for this paper from *Bio-protocol*.
