# Supplementary material for: Fatty acid desaturase 2 determines the lipidomic landscape and steroidogenic function of the adrenal gland
Source: Sci Adv. 2023 Jul 21;9(29):eadf6710. doi: 10.1126/sciadv.adf6710 (PMC10361602; doi:10.1126/sciadv.adf6710)
Supplement: Supplementary file 1 — Tables S1 to S6 Figs. S1 to S6 [file sciadv.adf6710_sm.pdf]

Science Advances
AAAS

Supplementary Materials for

Fatty acid desaturase 2 determines the lipidomic landscape and steroidogenic function of the adrenal gland

Anke Witt *et al.*

Corresponding author: Vasileia Ismini Alexaki, vasileiaIsmini.alexaki@uniklinikum-dresden.de

Sci. Adv. **9**, eadf6710 (2023)
DOI: 10.1126/sciadv.adf6710

This PDF file includes:

Tables S1 to S6
Figs. S1 to S6

Supplemental Tables

Table S1. Lipidome composition of adrenal glands of LFD- and HFD-fed mice

	Storage lipids (% mean ± SD)	Non-storage lipids (% mean ± SD)
**LFD**	86.98 ± 5.43	13.02 ± 5.22
**HFD**	90.12 ± 1.93	9.87 ± 1.89

Table S2. Lipid class abundance in adrenal glands of LFD- and HFD-fed mice

pmol/µg protein (Aver ± SD)	LFD	HFD	Stat.
**CE**	246.59 ± 68.57	280.49 ± 41.86	ns
**Ceramides**	0.86 ± 0.16	1.27 ± 0.35	ns
**Cholesterol**	37.03 ± 5.65	37.12 ± 3.64	ns
**CL**	1.35 ± 0.27	1.44 ± 0.31	ns
**DAG**	12.84 ± 8.06	10.26 ± 3.26	ns
**HexCer**	0.0781 ± 0.0186	0.0646 ± 0.0089	ns
**LPA**	0.00430 ± 0.00398	0.00628 ± 0.00148	ns
**LPC**	3.98 ± 1.25	3.49 ± 0.47	ns
**LPE**	0.91 ± 0.2	0.84 ± 0.11	ns
**LPE O-**	0.33 ± 0.12	0.28 ± 0.05	ns
**LPG**	0.0171 ± 0.0053	0.0246 ± 0.0075	ns
**LPI**	0.0876 ± 0.0123	0.1330 ± 0.0188	ns
**LPS**	0.0287 ± 0.0139	0.0395 ± 0.0052	ns
**PA**	0.0198 ± 0.0200	0.0304 ± 0.0149	ns
**PC**	68.54 ± 6.82	70.56 ± 12.32	ns
**PC O-**	2.07 ± 0.15	1.95 ± 0.27	ns
**PE**	24.72 ± 5.09	29.84 ± 7.06	ns
**PE O-**	18.23 ± 2.49	17.84 ± 1.65	ns
**PG**	0.869 ± 0.067	0.766 ± 0.406	ns
**PI**	12.15 ± 1.94	13.3 ± 2.76	ns
**PS**	5.9 ± 0.49	5.73 ± 0.72	ns
**SM**	5.29 ± 0.75	5.56 ± 0.75	ns
**TAG**	1306.32 ± 767.16	1583.34 ± 338.58	ns
**Total**	3496.45 ± 1473.05	4128.73 ± 698.88	ns

ns: not significant

Table S3. Double bond index (DBI) and length index (LI) in storage and non-storage lipids in the adrenal glands of LFD- and HFD-fed mice

	STORAGE LIPIDS (Aver ± SD)			NON-STORAGE LIPIDS (Aver ± SD)		
	LFD	HFD	P-value	LFD	HFD	P-value
**DBI**	2.809 +/- 0.095	3.072 +/- 0.024	***	3.322 +/- 0.049	3.462 +/- 0.042	***
**LI**	45.291 +/- 4.203	47.537 +/- 1.223	ns	36.828 +/- 0.081	37.110 +/- 0.064	***

*** <0.001; ns: not significant

Table S4. Composition of diets used for feeding of *Fads2*$^{-/-}$ and WT mice

Fatty acid	PUFA-rich diet	Low-PUFA diet
**Total sat.**	**19.62 ± 0.03**	**47.39 ± 0.01**
**Total mono.**	**28.16 ± 0.07**	**38.02 ± 0.03**
18:2n-6	42.02 ± 0.03	9.27 ± 0.01
18:3n-6	0.02 ± 0.01	ND
20:2n-6	0.08 ± 0.001	ND
20:3n-6	0.02 ± 0.01	ND
20:4n-6	0.13 ± 0.003	ND
22:2n-6	ND	ND
22:4n-6	ND	ND
22:5n-6	ND	ND
**Total ω6 FAs**	**42.27 ± 0.03**	**9.27 ± 0.01**
18:3n-3	3.88 ± 0.02	2.43 ± 0.003
20:3n-3	ND	ND
20:5n-3	1.49 ± 0.01	ND
22:5n-3	0.25 ± 0.01	ND
22:6n-3	1.32 ± 0.01	ND
**Total ω3 FAs**	**6.25 ± 0.02**	**2.43 ± 0.003**
**Total FA (μg/mg)**	**47.00 ± 0.68**	**43.19 ± 1.15**

（% of total fatty acids; Mean ± SEM; ND, not detected）

Table S5. Lipidomics in adrenal glands of WT and *Fads2*$^{-/-}$ mice fed a PUFA-rich or low-PUFA diet

**Lipid species**	**Low-PUFA diet**			**PUFA-rich diet**		
	**WT (Aver±SD)**	***Fads2*$^{-/-}$ (Aver±SD)**	**P value**	**WT (Aver±SD)**	***Fads2*$^{-/-}$ (Aver±SD)**	**P value**
**C14:1 n-5**	0.033333±0.08165	0.083333 ± 0.132916	0.45057	n.d.	n.d.	n.a.
**C20:4 n-3 (ETA)**	0.95±0.207364	0.5±0.089443	0.00064 (***)	0.983333±0.263944	1.466667±0.338625	0.020215264 (*)
**C20:5 n-3 (EPA)**	41.6±9.324591	21.23333±2.881435	0.00046 (***)	45.71667±6.546882	66.2±17.14211	0.021037628 (*)
**C18:3 n-3 (ALA)**	18.93333±6.712575	27.81667±12.06887	0.14619	35.88333±24.94806	37.98333±6.767102	0.846254490

**C18:3 n-6 (GLA)**	6.483333±1.483801	4.383333±2.964737	0.15181	10.35±5.29783	6.283333±2.11605	0.111377473
**C14:0**	99.38333±16.63171	81.33333±4.507623	0.02809 (*)	59.05±33.32559	73.66667±11.16739	0.332368186
**C22:6 n-3 (DHA)**	1567.833±281.4653	765.5667±68.03622	0.00005 (****)	1911.067±374.2711	2059.133±373.6225	0.508415986
**C16:1 n-7**	163.3167±29.67716	163.2±19.59153	0.993746	83.81667±44.24249	91.2±13.55389	0.7041063
**C20:4 n-6 (ARA)**	7703.533±1051.61	1808.25±236.4437	0.0000001 (****)	7843.15±1187.82	6571.517±769.2027	0.0523427
**C18:2 n-6 (LA)**	650.3667±128.7274	1498.85±215.0335	0.000009 (****)	1208.883±386.0135	1592.8±208.8327	0.0577698
**C22:5 n-3 (DPA)**	175.6±27.43545	118.75±16.53273	0.00145 (**)	126.9±30.69111	157.1167±32.01777	0.1261116
**C22:5 n-6 (DPA)**	53.36667±7.226248	0.066667±0.163299	0.000000 (****)	16.18333±5.177419	n.d.	n.a.
**C20:3**	126.9667±18.00507	12.06667±5.333542	0.000000 (****)	155.1667±16.56365	31.21667±3.727421	0.0000000 (****)
**C16:0 (Palmitic acid,)**	1049.033±137.3459	1087.883±196.4046	0.699660	791.6833±295.7263	1176.983±237.1646	0.0320074 (*)
**C18:1 n-9 cis (OA)**	1754±285.4089	2170.517±221.2694	0.0180016 (*)	1056.183±308.9346	1199.933±136.9281	0.3219459
**C18:1 n-9 trans (EA)**	1887.533±276.9233	2223.267±222.4705	0.043129 (*)	1227.933±321.9364	1330.717±127.5553	0.48386794
**C20:2 n-6 (DGLA)**	28.18333±4.257895	136.45±19.96034	0.0000001 (****)	41.78333±6.146029	79.81667±19.75605	0.00113822 (**)
**C18:0 (Stearic acid)**	693.7333±104.6519	657.9167±96.40173	0.55128	580.9833±141.9845	730.2833±127.7511	0.0845414
**C20:1 n-9**	75.58333±15.21084	162.6±24.82402	0.000025 (****)	29.85±11.24736	40.66667±7.336939	0.0767587
**C22:2 n-6**	n.d.	0.9±0.745654	n.a.	n.d.	0.183333±0.213698	n.a.
**C22:1 n-9**	18.65±1.615859	34.36667±9.332452	0.002269 (**)	n.d.	24.96667±5.699006	n.a.
**C22:0**	254.65±112.2897	269.2167±129.8201	0.8394989	349.5167±219.8333	261.0667±112.668	0.40102768

* <0.05, ** <0.01, *** <0.001, **** <0.0001; n.d.: not detected, n.a.: not applicable

Table S6. List of primers

*Cyp11a1*_Fwd	5'GCTGAGTACTGGAAAGGGAGC3'
*Cyp11a1*_Rev	5'TGCCCAGCTTCTCCCTGTAA3'
*Cyp11b1*_Fwd	5'ATAGAGAACTCCGTGGCCTG3'
*Cyp11b1*_Rev	5'TGCAGTCGGTTGAAGTACCA3'

*Cyp11b2*_Fwd	5’AGAATGGCGTCTCAACCGAC3’
*Cyp11b2*_Rev	5’AGTCCCTTGCTACCATGTCC3’
*Cyp21a1*_Fwd	5’GCTGTGGCTTTCCTGCTTCAC3’
*Cyp21a1*_Rev	5’GGCCCAGCTTGAGGTCTAACT3’
*Fads2*_Fwd	5’TCCTCTCGTACTTCGGCACT3’
*Fads2*_Rev	5’GGTTCCACCAGTTGGCTGAG3’
*3β-Hsd2*_Fwd	5’AGGGAGCTCTCAATTGTGCC3’
*3β-Hsd2*_Rev	5’GCTTAGAAAGGCTGGTTCTGG3’
*Star*_Fwd	5’CTGTCCACCACATTGACCTG3’
*Star*_Rev	5’CAGCTATGCAGTGGGAGACA3’
*Tbp*_Fwd	5’AGAACAATCCAGACTAGCAGCA3’
*Tbp*_Rev	5’GGGAACTTCACATCACAGCTC3’
Mm *18S* Fwd	5’GTTCCGACCATAAACGATGCC3’
Mm *18S* Rev	5’TGGTGGTGCCCTTCCGTCAAT3’
FADS2 Fwd	5’CACGGCCTTTGTCCTTGCTA3’
FADS2 Rev	5’AGATGTTAGGCTTGGCGTGG3’
STAR Fwd	5’GGGAGTGGAACCCCAATGTC3’
STAR Rev	5’CCAGCTCGTGAGTAATGAATGT3’
Hs *18S* Fwd	5’TGCCCTATCAACTTTCGATG3’
Hs *18S* Rev	5’GATGTGGTAGCCGTTTCTCA3’

Supplemental Figure legends

Figure S1. FADS2 is required for steroidogenesis in adrenocortical cells

A. FADS2 protein expression in liver (Liv), adrenal glands (Adr), kidney (Kid), brain (Br) and gonadal adipose tissue (GAT) in two wild-type mice (WT1, WT2) assessed by western blot using α-Tubulin as a loading control. Adrenal glands of *Fads2*$^{-/-}$ mice ($KO_{adrenal}$) were used as negative control. **B.** PUFA synthesis pathway. **C.** Western blot of cell lysate fractions from NCI-H295R cells, separated by Optiprep density gradient. Same amount of protein from all fractions was blotted per lane. Representative blots are shown. Fractions 10-12 (SDHB positive) were identified as mitochondrial, fractions 4-5 (HSL positive) were identified as lipid droplets. **D.** Cholesterol and CE content in the lipid droplet fractions of NCI-H295R cells treated for 18 h with SC-26196 (10 µM) or DMSO (Ctrl); results are shown as fold increase in SC-26196-treated compared to DMSO-treated cells. Results of one out of two experiments are shown. **E.** Cell viability of primary adrenal cells treated for 18 h with SC-26196 (10 µM) or DMSO (n=4). **F.** NCI-H295R cells were treated with SC-26196 (10 µM) or DMSO (Ctrl) for 18 h followed by 30 min or 24 h treatment with forskolin (FSK, 1 µM), in presence of SC-26196 or DMSO. Steroids were measured in the cell culture supernatants by LC-MS/MS. **G.** Relative expression of *Star, Cyp11a1, 3β-Hsd2, Cyp11b1* and *Cyp11b2* in primary adrenal cell cultures treated for 18 h with SC-26196 (10 µM) or DMSO (Ctrl). *Tbp* expression was used as a housekeeping gene. Relative gene expression of DMSO-treated cells was set to 1 (n=11). **H.** *FADS2* expression in NCI-H295R cells transfected for 24 h with a FADS2-overexpressing plasmid (pFADS2) or a control plasmid (pCMV); *18S* expression was used as a housekeeping gene (n=4). **I.** FADS2 expression in pFADS2- or pCMV-transfected NCI-H295R cells assessed by western blot (24 h post-transfection). Vinculin was used as a loading control. **J.** 11-Deoxycorticosterone, cortisol and aldosterone levels were measured by LC-MS/MS in cell culture supernatants of pFADS2- or pCMV-transfected NCI-H295R cells treated 24 h post-transfection for 24 h with forskolin (FSK, 1 µM) (n=5-6). Data in **E-H** and **J** are shown as mean ± SEM; * p-value<0.05, ** p-value<0.01, *** p-value<0.001; **** p-value<0.0001

Figure S2. Obesity-related changes in lipid composition and gene transcription in the adrenal gland

A. ACTH plasma levels in LFD and HFD mice (n=6-8). **B.** Adrenal gland weight in LFD and HFD mice (7-10). Data in **A** and **B** are shown as mean ± SEM; * p-value<0.05 **C.** FADS2 protein expression in liver and brain of LFD and HFD mice (n=5). Liver of *Fads2*$^{-/-}$ mice (KO_{liver}) was used as negative control, α-Tubulin was used as a loading control. **D.** Principal component analysis (PCA) performed on all lipids, non-storage and storage lipids in adrenal glands of mice fed for 20 weeks a LFD or HFD. **E-H.** Storage (CE, TAG) (**E**) and non-storage lipids (phospholipids, lysophospholipids and sphingolipids; cholesterol was excluded from the calculation) (**G**) in adrenal glands of mice fed a LFD or HFD were regrouped according to the number of carbon atoms of the acyl chains; only the length of groups with a mean abundance > 1 mol % are shown. Storage (**F**) and non-storage lipids (**H**) were regrouped according to the number of double bonds of their acyl chains. **I,J.** PC (**I**) and PE (**J**) lipid species in adrenal glands of lean and obese mice. Only the species

with a mean abundance > 1 mol % are shown. The mean mol % ± standard deviation (SD) are shown, (n=7-8 mice per group); * p-value<0.05; ** p-value<0.01; *** p-value<0.001. **K-P.** GSEA based on RNA-Seq in the adrenal cortex of mice fed for 20 weeks a LFD or HFD for genes involved in metabolism of lipids (**K**), mitochondrial biogenesis (**L**), respiratory electron transport (**M**), oxidative phosphorylation (**N**), citrate cycle (**O**) and fatty acid beta oxidation (**P**) (n=4 mice per group).

Figure S3. FADS2 inhibition reduces corticosteroid levels in obese mice

A. 11-Dehydrocorticosterone levels in hair of HFD mice 3 weeks after SC-26196 treatment start (n=8-10 mice per group). **B.** Glucose tolerance test in HFD mice performed at feeding week 14 (n=8-10 mice per group). Data are shown as mean ± SEM; ** p-value<0.01.

Figure S4. Diet supplementation with icosapent ethyl transcriptionally reprograms mitochondrial metabolism in the adrenal cortex and does not affect ACTH levels

A-C. Body weight (**A**), glucose tolerance test (**B**) and insulin tolerance test (**C**) in mice receiving a LFD, a HFD of a HFD with icosapent ethyl (n=8 mice per group). **D-F.** GSEA based on RNA-Seq in the adrenal cortex of mice fed a HFD supplemented with icosapent ethyl versus mice fed a HFD without icosapent ethyl for genes related to respiratory electron transport (**D**), oxidative phosphorylation (**E**) and citrate cycle (**F**) (n=4 mice per group). **G.** ACTH measurement in plasma of mice fed a HFD containing or not icosapent ethyl (n=8 mice per group). Data in A-C and G are shown as mean ± SEM.

Figure S5. FADS2 deficiency does not affect adrenal gland size, ACTH levels or steroidogenic enzyme expression

A. Hematoxylin and eosin staining in adrenal glands of WT and *Fads2*$^{-/-}$ mice kept under a PUFA-rich or a low-PUFA diet. Representative photos of one caption (out of 7-8) per adrenal gland of WT and *Fads2*$^{-/-}$ mice are shown (n=5 mice per group). Scale bar, 200 µm **B.** ACTH measurement in plasma of WT and *Fads2*$^{-/-}$ mice fed a PUFA-rich or low-PUFA diet (n=6-9 mice per group). **C.** Relative *Cyp11a1*, *3β-Hsd2*, *Cyp11b1* and *Cyp11b2* expression in the adrenal glands of WT and *Fads2*$^{-/-}$ mice fed a PUFA-rich or low-PUFA diet. Gene expression was set as 1 in WT mice fed a low-PUFA diet. *Tbp* was used as housekeeping gene (n=3 mice per group). **D.** Representative electron microscopy images of adrenocortical cells of WT and *Fads2*$^{-/-}$ mice fed a PUFA-rich or low-PUFA diet (magnification 1,200x). Adrenal glands of two WT and two *Fads2*$^{-/-}$ mice fed a low-PUFA and one WT and one *Fads2*$^{-/-}$ mice fed a PUFA-rich diet were examined. Asterisks (*) depict lipid droplets. Scale bar, 1 µm Data in **B** and **C** are shown as mean ± SEM.

Figure S6. *FADS2* expression correlates with expression of steroidogenic genes in the human adrenal gland

A-G. Correlation of *FADS2* expression with the expression of *STAR* (**A**), *VDAC1* (**B**), *CYP11A1* (**C**), *SF-1* (**D**) and *PRKACA* (**E**) in the human adrenal gland (**A-E**) and with the expression *SF-1* (**F**) and *PRKACA* (**G**)

in adrenocortical carcinoma (**F,G**), based on data from the public GEPIA2 database (http://gepia2.cancer-pku.cn/#index).

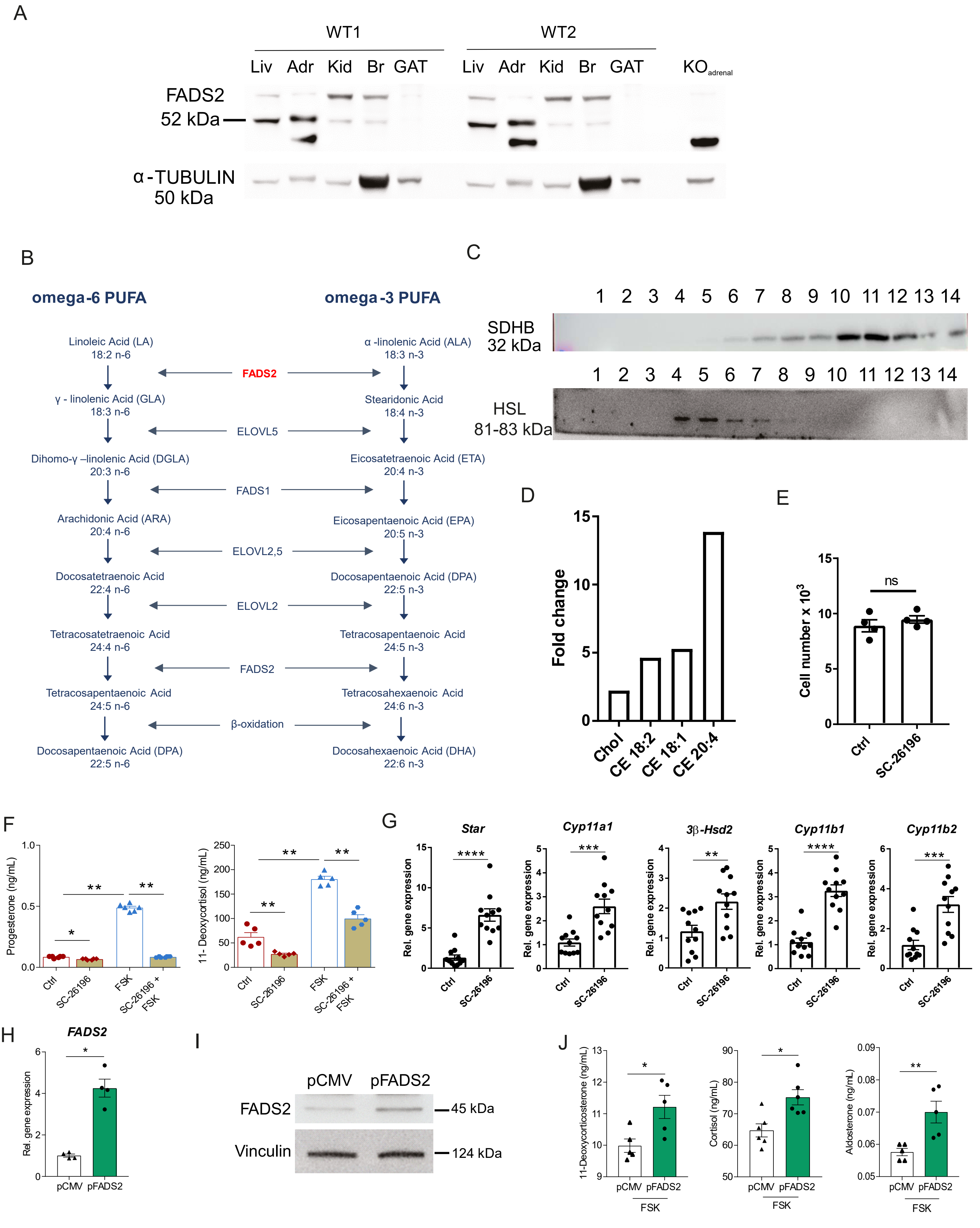
A
WT1
WT2
Liv Adr Kid Br GAT
Liv Adr Kid Br GAT
KOadrenal
FADS2
52 kDa
α-TUBULIN
50 kDa
B
omega-6 PUFA
omega-3 PUFA
Linoleic Acid (LA) 18:2 n-6
α-linolenic Acid (ALA) 18:3 n-3
FADS2
γ-linolenic Acid (GLA) 18:3 n-6
Stearidonic Acid 18:4 n-3
ELOVL5
Dihomo-γ-linolenic Acid (DGLA) 20:3 n-6
Eicosatetraenoic Acid (ETA) 20:4 n-3
FADS1
Arachidonic Acid (ARA) 20:4 n-6
Eicosapentaenoic Acid (EPA) 20:5 n-3
ELOVL2,5
Docosatetraenoic Acid 22:4 n-6
Docosapentaenoic Acid (DPA) 22:5 n-3
ELOVL2
Tetracosatetraenoic Acid 24:4 n-6
Tetracosapentaenoic Acid 24:5 n-3
FADS2
Tetracosapentaenoic Acid 24:5 n-6
Tetracosahexaenoic Acid 24:6 n-3
β-oxidation
Docosapentaenoic Acid (DPA) 22:5 n-6
Docosahexaenoic Acid (DHA) 22:6 n-3
C
1 2 3 4 5 6 7 8 9 10 11 12 13 14
SDHB
32 kDa
HSL
81-83 kDa
D
Fold change
Chol
CE 18:2
CE 18:1
CE 20:4
E
Cell number x 10³
ns
Ctrl
SC-26196
F
Progesterone (ng/mL)
11- Deoxycortisol (ng/mL)
Ctrl
SC-26196
FSK
SC-26196 + FSK
G
Star
Cyp11a1
3β-Hsd2
Cyp11b1
Cyp11b2
Rel. gene expression
H
FADS2
Rel. gene expression
pCMV
pFADS2
I
pCMV
pFADS2
FADS2
Vinculin
45 kDa
124 kDa
J
11-Deoxycorticosterone (ng/mL)
Cortisol (ng/mL)
Aldosterone (ng/mL)
pCMV
pFADS2
FSK

Figure S1

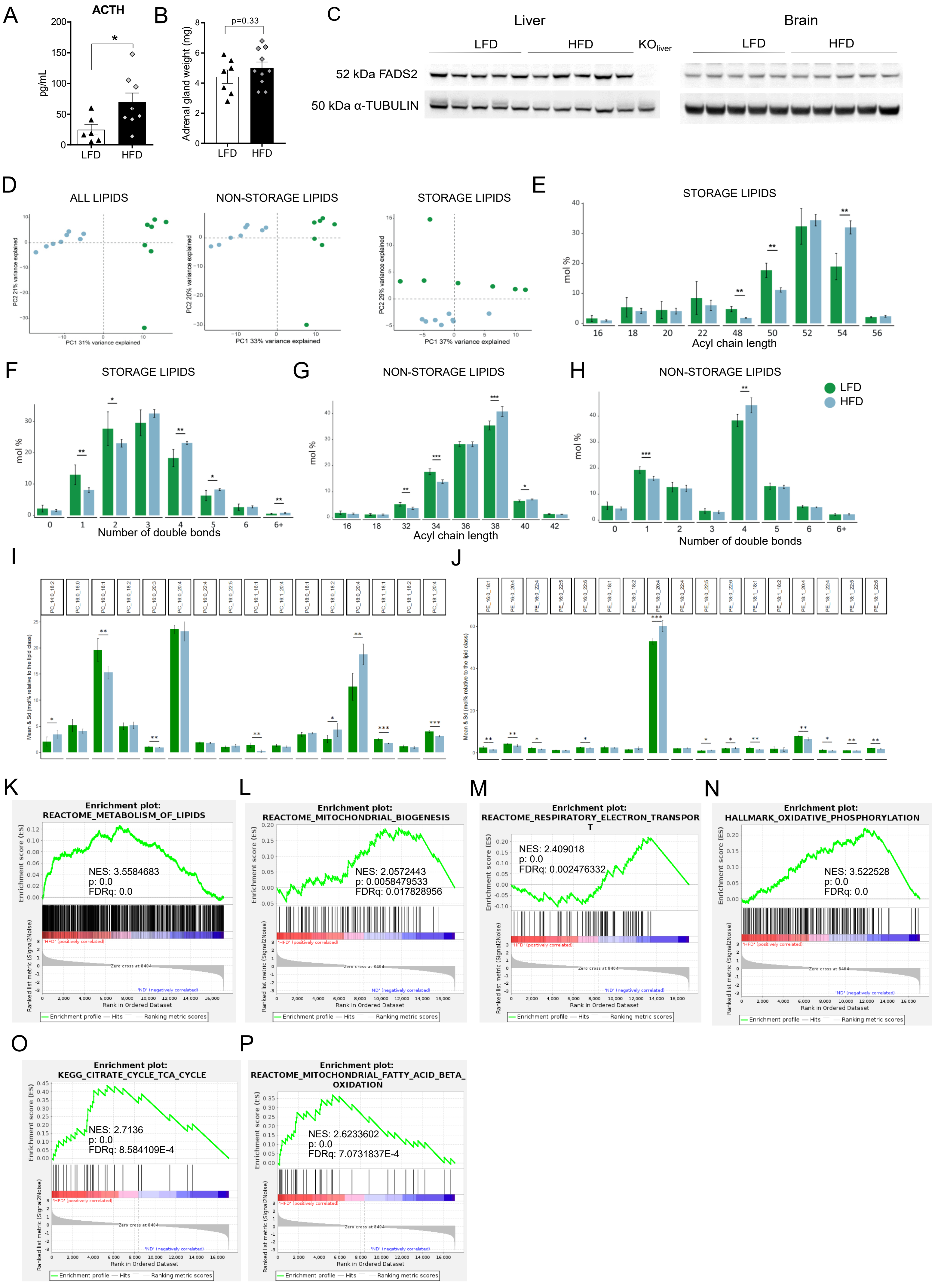
A
ACTH
pg/mL
LFD
HFD
B
p=0.33
Adrenal gland weight (mg)
LFD
HFD
C
Liver
Brain
LFD
HFD
KOliver
52 kDa FADS2
50 kDa α-TUBULIN
D
ALL LIPIDS
NON-STORAGE LIPIDS
STORAGE LIPIDS
PC1 31% variance explained
PC2 21% variance explained
PC1 33% variance explained
PC2 20% variance explained
PC1 37% variance explained
PC2 29% variance explained
E
STORAGE LIPIDS
mol %
Acyl chain length
F
STORAGE LIPIDS
Number of double bonds
G
NON-STORAGE LIPIDS
Acyl chain length
H
NON-STORAGE LIPIDS
Number of double bonds
LFD
HFD
I
J
Mean & Sd (mol% relative to the lipid class)
K
Enrichment plot:
REACTOME_METABOLISM_OF_LIPIDS
NES: 3.5584683
p: 0.0
FDRq: 0.0
L
Enrichment plot:
REACTOME_MITOCHONDRIAL_BIOGENESIS
NES: 2.0572443
p: 0.0058479533
FDRq: 0.017828956
M
Enrichment plot:
REACTOME_RESPIRATORY_ELECTRON_TRANSPORT
NES: 2.409018
p: 0.0
FDRq: 0.002476332
N
Enrichment plot:
HALLMARK_OXIDATIVE_PHOSPHORYLATION
NES: 3.522528
p: 0.0
FDRq: 0.0
O
Enrichment plot:
KEGG_CITRATE_CYCLE_TCA_CYCLE
NES: 2.7136
p: 0.0
FDRq: 8.584109E-4
P
Enrichment plot:
REACTOME_MITOCHONDRIAL_FATTY_ACID_BETA_OXIDATION
NES: 2.6233602
p: 0.0
FDRq: 7.0731837E-4
Enrichment score (ES)
Ranked list metric (Signal2Noise)
Rank in Ordered Dataset
Enrichment profile — Hits — Ranking metric scores

Figure S2

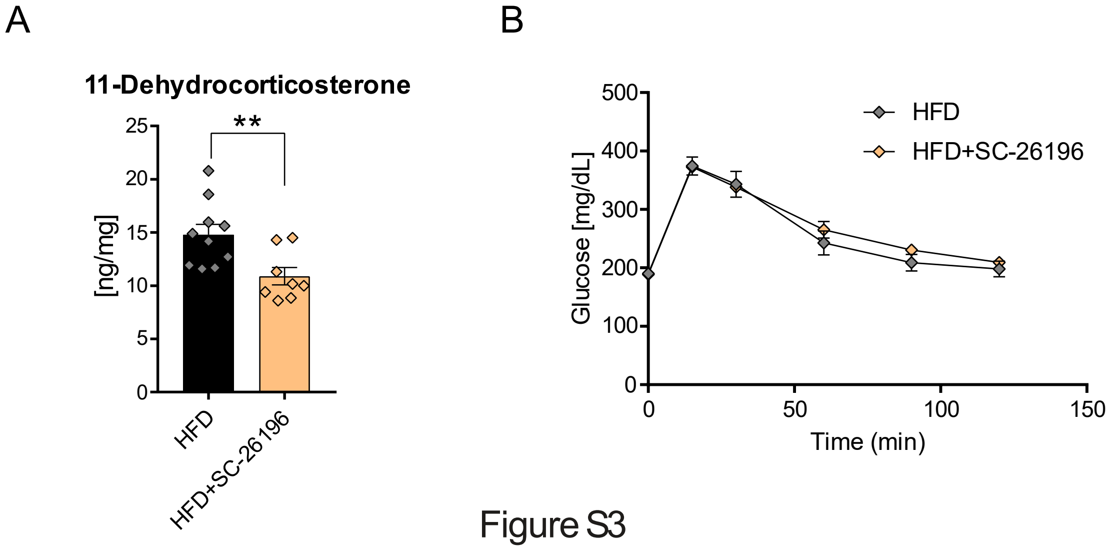
A
11-Dehydrocorticosterone
**
[ng/mg]
0
5
10
15
20
25
HFD
HFD+SC-26196
B
HFD
HFD+SC-26196
Glucose [mg/dL]
0
100
200
300
400
500
0
50
100
150
Time (min)

Figure S3

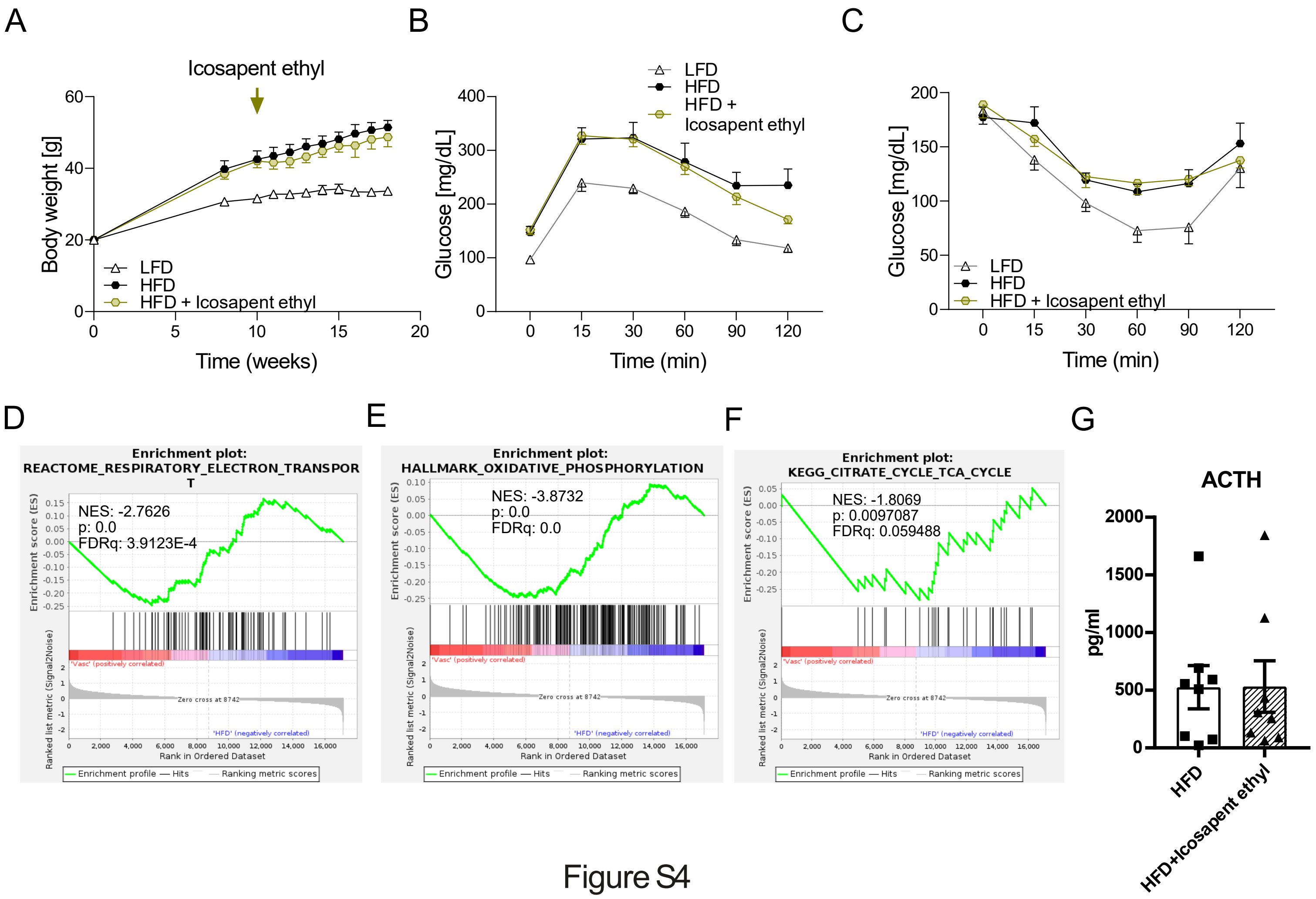
A
Icosapent ethyl
Body weight [g]
Time (weeks)
LFD
HFD
HFD + Icosapent ethyl
B
Glucose [mg/dL]
Time (min)
LFD
HFD
HFD + Icosapent ethyl
C
Glucose [mg/dL]
Time (min)
LFD
HFD
HFD + Icosapent ethyl
D
Enrichment plot: REACTOME_RESPIRATORY_ELECTRON_TRANSPORT
NES: -2.7626
p: 0.0
FDRq: 3.9123E-4
E
Enrichment plot: HALLMARK_OXIDATIVE_PHOSPHORYLATION
NES: -3.8732
p: 0.0
FDRq: 0.0
F
Enrichment plot: KEGG_CITRATE_CYCLE_TCA_CYCLE
NES: -1.8069
p: 0.0097087
FDRq: 0.059488
Enrichment score (ES)
Ranked list metric (Signal2Noise)
'Vasc' (positively correlated)
'HFD' (negatively correlated)
Zero cross at 8742
Rank in Ordered Dataset
Enrichment profile — Hits
Ranking metric scores
G
ACTH
pg/ml
HFD
HFD+Icosapent ethyl

Figure S4

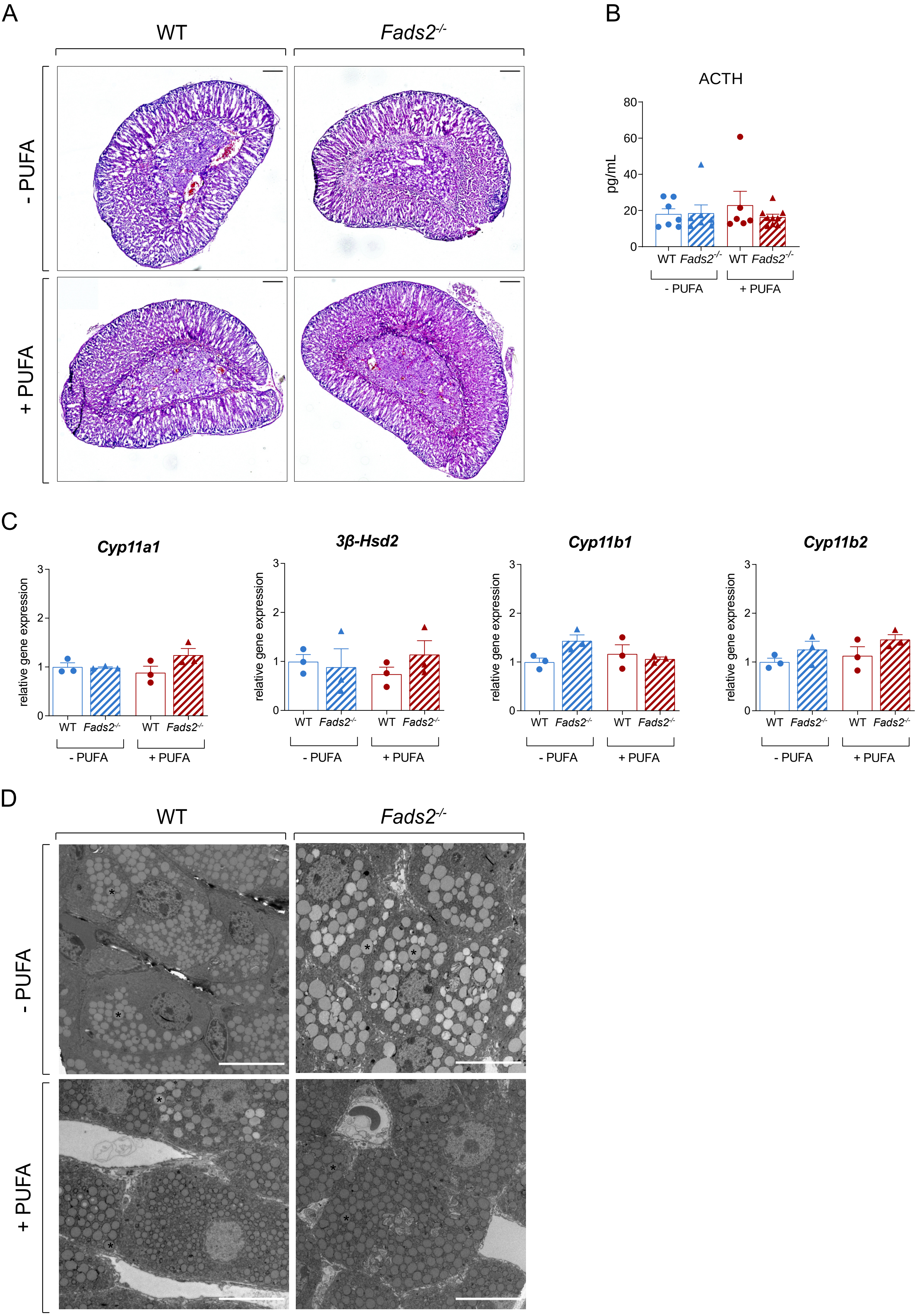
A
WT
Fads2-/-
- PUFA
+ PUFA
B
ACTH
pg/mL
80
60
40
20
0
WT Fads2-/- WT Fads2-/-
- PUFA
+ PUFA
C
Cyp11a1
3β-Hsd2
Cyp11b1
Cyp11b2
relative gene expression
3
2
1
0
WT Fads2-/- WT Fads2-/-
- PUFA
+ PUFA
D
WT
Fads2-/-
- PUFA
+ PUFA

Figure S5

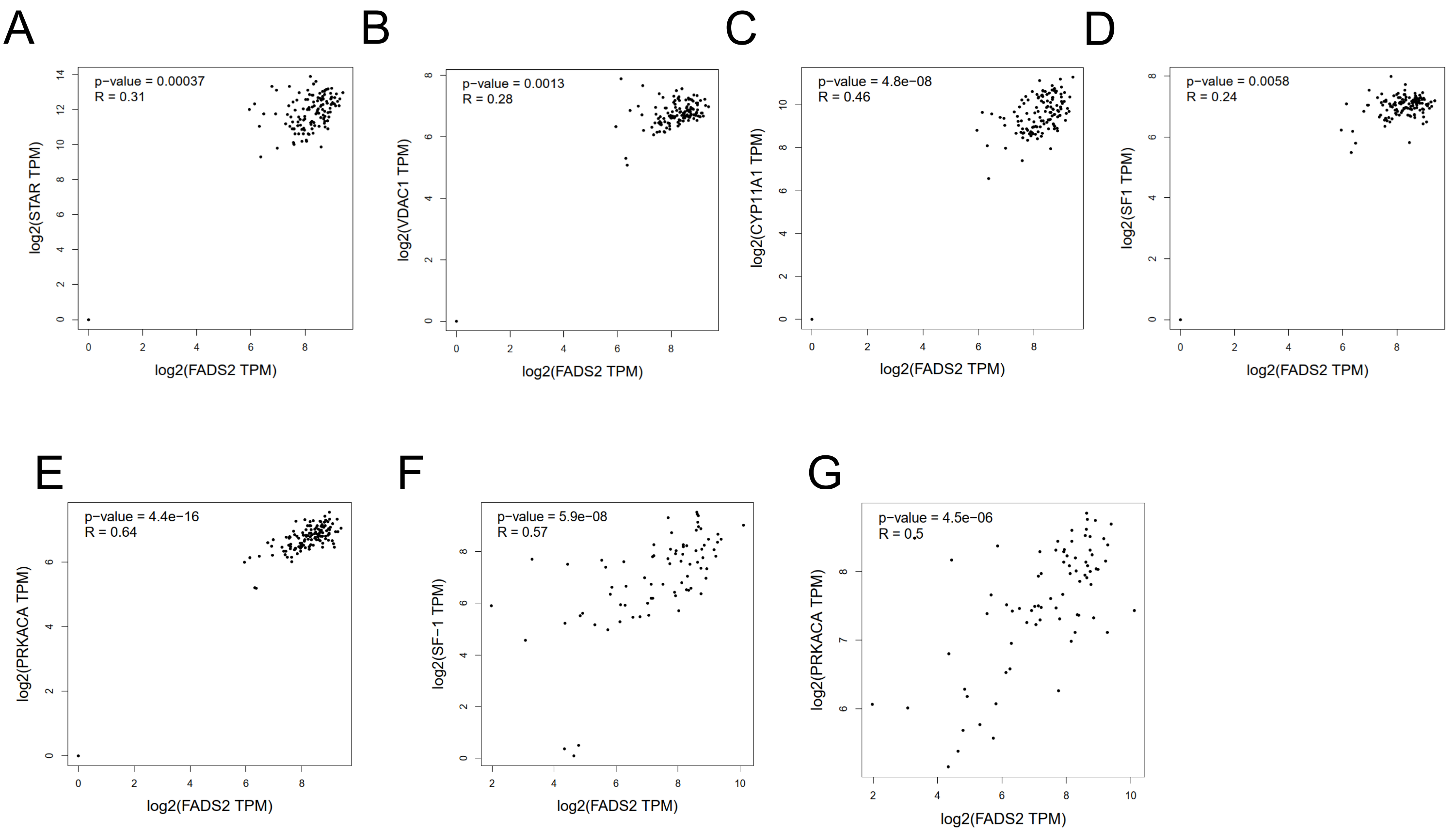
A
p−value = 0.00037
R = 0.31
log2(STAR TPM)
log2(FADS2 TPM)
B
p−value = 0.0013
R = 0.28
log2(VDAC1 TPM)
log2(FADS2 TPM)
C
p−value = 4.8e−08
R = 0.46
log2(CYP11A1 TPM)
log2(FADS2 TPM)
D
p−value = 0.0058
R = 0.24
log2(SF1 TPM)
log2(FADS2 TPM)
E
p−value = 4.4e−16
R = 0.64
log2(PRKACA TPM)
log2(FADS2 TPM)
F
p−value = 5.9e−08
R = 0.57
log2(SF−1 TPM)
log2(FADS2 TPM)
G
p−value = 4.5e−06
R = 0.5
log2(PRKACA TPM)
log2(FADS2 TPM)

Figure S6
